# Many continuous variables should be analyzed using the relative scale: a case study of β_2_-agonists for preventing exercise-induced bronchoconstriction

**DOI:** 10.1186/s13643-019-1183-5

**Published:** 2019-11-19

**Authors:** Harri Hemilä, Jan O. Friedrich

**Affiliations:** 1 Department of Public Health, POB 20 University of Helsinki, Tukholmankatu 8 B, FI-00014 Helsinki, Finland; 2 Critical Care and Medicine Departments and Li Ka Shing Knowledge Institute, University of Toronto and St. Michael’s Hospital, Toronto, Canada

**Keywords:** Adrenergic beta-2 receptor agonists, Albuterol, Ecological fallacy, Exercise-induced asthma, Forced expiratory volume, Meta-analysis, Outcome assessment, Randomized controlled trial, Spirometry, Statistics

## Abstract

**Background:**

The relative scale adjusts for baseline variability and therefore may lead to findings that can be generalized more widely. It is routinely used for the analysis of binary outcomes but only rarely for continuous outcomes. Our objective was to compare relative vs absolute scale pooled outcomes using data from a recently published Cochrane systematic review that reported only absolute effects of inhaled β_2_-agonists on exercise-induced decline in forced-expiratory volumes in 1 s (FEV_1_).

**Methods:**

From the Cochrane review, we selected placebo-controlled cross-over studies that reported individual participant data (IPD). Reversal in FEV_1_ decline after exercise was modeled as a mean uniform percentage point (pp) change (absolute effect) or average percent change (relative effect) using either intercept-only or slope-only, respectively, linear mixed-effect models. We also calculated the pooled relative effect estimates using standard random-effects, inverse-variance-weighting meta-analysis using study-level mean effects.

**Results:**

Fourteen studies with 187 participants were identified for the IPD analysis. On the absolute scale, β_2_-agonists decreased the exercise-induced FEV_1_ decline by 28 pp., and on the relative scale, they decreased the FEV_1_ decline by 90%. The fit of the statistical model was significantly better with the relative 90% estimate compared with the absolute 28 pp. estimate. Furthermore, the median residuals (5.8 vs. 10.8 pp) were substantially smaller in the relative effect model than in the absolute effect model. Using standard study-level meta-analysis of the same 14 studies, β_2_-agonists reduced exercise-induced FEV_1_ decline on the relative scale by a similar amount: 83% or 90%, depending on the method of calculating the relative effect.

**Conclusions:**

Compared with the absolute scale, the relative scale captures more effectively the variation in the effects of β_2_-agonists on exercise-induced FEV_1_-declines. The absolute scale has been used in the analysis of FEV_1_ changes and may have led to sub-optimal statistical analysis in some cases. The choice between the absolute and relative scale should be determined based on biological reasoning and empirical testing to identify the scale that leads to lower heterogeneity.

## Background

The relative scale has been used for decades for estimating the effects on binary outcomes, such as calculating that heavy alcohol consumption increases the occurrence of liver cirrhosis by the rate ratio (RR) of over 10 [1]. It is also standard for survival analysis, using hazard ratios, and for comparisons of incidence rates, using incidence rate ratios. Meta-analyses have shown that the relative scale leads to less heterogeneity in the analysis of binary outcomes compared with the absolute scale (i.e. rate differences), which indicates that the relative scale better captures the biological effects [2]. In contrast, the relative scale has rarely been used in the meta-analysis of continuous outcomes and it is not available as an option in popular meta-analysis software such as the RevMan program of the Cochrane collaboration [3]. Instead, meta-analyses of continuous outcomes typically use the absolute scale, i.e., the original measurement units (mean difference, MD), or the standardized mean difference (SMD) scale in which the mean difference is expressed in the pooled standard deviation units. Both of these approaches (MD and SMD) are available as options in popular meta-analysis software [3].

The selection of scale for continuous outcomes is relevant in the analysis of a single trial and in the meta-analysis of several trials. In a single trial, the scale influences the interpretation of the findings and the communication between researchers, clinicians and patients [4]. In the case of a meta-analysis, the scale additionally influences the comparability of the trials, namely, the relative scale adjusts for the baseline variability in continuous outcomes in the same sense as the pooled RR adjusts for the baseline variability in risk between different studies in the analysis of binary outcomes. In meta-analyses that pooled diverse research topics of continuous outcomes, heterogeneity was less on the relative scale, than on the absolute scale [5–7]. This suggests that the relative scale may better capture also many biological effects that are measured using continuous outcomes. As one illustration, the relative scale was demonstrated to be more informative in the analysis of disease duration compared with using the MD scale [8–10].

The current study was motivated by the Cochrane review by Bonini et al., which examined the effects of β_2_-agonists on exercise-induced bronchoconstriction (EIB) [11]. The usual limit for classifying that a person has the condition EIB is a ≥ 10% decline in forced expiratory volume in 1 s (FEV_1_) in a standardized exercise test [12]. Based on 72 comparisons from 44 studies, Bonini et al. calculated that β_2_-agonists reduced the exercise-induced FEV_1_ decline by 17.67 percentage points (pp) (95% CI: 15.84 to 19.51 pp) [11]. However, one person may suffer from an 11% decline in FEV_1_ by exercise and another person may suffer from an 80% decline in FEV_1_, yet both of them are similarly classified as cases of EIB. The Cochrane review implies that the expected effect of 17.67 pp. reduction in exercise-induced FEV_1_ decline applies for both persons. However, it seems likely that the former person has an effect of β_2_-agonist much less, whereas the latter person might have an effect much greater than the overall mean of 17.67 pp. reduction in FEV_1_ decline.

The β_2_-agonists were invented in the middle of the 1900s and their efficacy against EIB was demonstrated in numerous clinical trials starting from the 1970s [12–16]. Thus, it is not relevant to ask the null hypothesis type of question whether β_2_-agonists differ from placebo in their influences on EIB. Instead, the important question is to estimate the average size of the effect and the variation in effect size between individuals.

The goal of this study was to compare the usefulness of the relative and the absolute scales in the estimation of the effects of β_2_-agonists on exercise-induced FEV_1_ decline. If the relative scale better captures the effects of interventions on FEV_1_ changes, then the meta-analyses that have used an absolute scale such as MD for analyzing the effects on FEV_1_ changes [11] may have led to sub-optimal estimates.

## Methods

### Selection of the β_2_-agonist trials on EIB

No new literature search was done for this analysis, since Bonini et al. [11] carried out recently a thorough search of the literature on controlled trials of β_2_-agonists for EIB.

For the independent participant data (IPD) analysis, we systematically reviewed all the included and excluded studies, and their reference lists in the studies identified by Bonini et al. [11], and included all placebo-controlled inhalatory β_2_-agonist cross-over randomized trials that reported IPD, 13 trials [17–29]. Bonini et al. excluded trials for a few reasons, one being “no clear diagnosis of exercise-induced bronchoconstriction”. We did not exclude such trials for the following reasons: clear dichotomous definition of EIB, such as a ≥ 10% or a ≥ 15% FEV_1_ decline in an exercise test [12] is relevant in certain contexts such as in top level athletics; however, such a cut-off level is arbitrary and has no biological basis, and dichotomization of continuous variables decreases statistical power. Moreover, if participants with small FEV_1_ declines are included in the analysis, the range of FEV_1_ declines becomes wider and the comparison of the absolute scale (intercept) and the relative scale (slope) becomes statistically more powerful. One trial that was excluded by Bonini et al. on the basis that there was no clear diagnosis of EIB reported IPD for exercise-induced FEV_1_ decline and was included in our IPD analysis [22]. Another trial with IPD data was identified through perusal of the reference lists in included RCTs, and was included in our analysis [30], but had not been identified by Bonini [11]. Thus, a total of 14 trials reporting IPD suitable for this analysis were identified (Table 1).

**Table 1:** Characteristics of included trials with IPD

Trial (year)[ref.]	*N*	β_2_-Agonist
Anderson (2001) [17]	27	Salbutamol
Boner (1994) [18]	15	Salbutamol
de Benedictis (1998) [19]	12	Salbutamol
Henriksen (1992) [20]	12	Salbutamol
Pearlman (2007) [21]	15	Salbutamol
Robertson (1994) [22]	8	Salbutamol
de Benedictis (1996) [23]	12	Salmeterol
Green (1992) [24]	13	Salmeterol
Simons (1997) [25]	11	Salmeterol
Dinh Xuan (1989) [26]	10	Terbutaline
Henriksen (1983) [27]	14	Terbutaline
Debelic (1988) [28]	16	Reproterol
Walker (1986) [29]	12	Bitolterol
Schoeffel (1981) [30]	10	Metaproterenol
All IPD trials	187	

For the study-level linear mixed-effect models, we included all the 44 cross-over trials that were included in the Analysis 1.1 of Bonini [11]. The characteristics of the 44 trials were described previously [11], and are not summarized in this report. For the standard study-level meta-analyses, we included the 14 trials that reported IPD.

### Extraction of data

When several β_2_-agonists were investigated in the same report, we selected salbutamol if that was used; and if not, salmeterol, in an attempt to decrease the heterogeneity of the comparisons. When exercise tests were repeated several times after the administration of a β_2_-agonist, we selected the shortest delay between the β_2_-agonist administration and the exercise test. In some trials, β_2_-agonists were administered for several days or weeks, and we selected the shortest administration before the exercise test. There have been discussions about whether the most appropriate baseline in an EIB study is before drug administration (pre-drug) or after drug administration (post-drug) [31, 32]. In cases when both levels were available, we selected the pre-drug level as the baseline.

For the IPD analysis, individual exercise-induced FEV_1_ declines were extracted from the 14 trial reports. Two studies reported IPD results as figures and the FEV_1_ declines were measured from them [25, 26]. For the study-level mixed-effect model analysis of the 44 trials, we extracted the numerical values for FEV_1_ declines from the reports, or measured the mean FEV_1_ declines from published figures when numerical data were not published. See Additional file 1: Table S1 and Table S2 for description of the details in the selections of the IPD and study means, and see Additional file 2 for the extracted data. Some inaccuracies and errors in data extraction in Bonini et al. [11] were identified and corrected if required, see Additional file 1: Table S4.

### Statistical methods

**Fig. 1:**
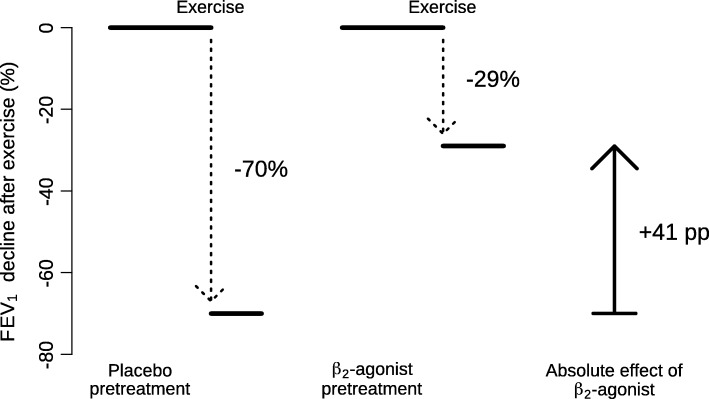
Description of the pulmonary function measurements on which the IPD analysis is based. The exercise-induced decline in FEV_1_ was measured on two occasions: after the administration of placebo and after the β_2_-agonist. The figures are for the participant X shown in Fig. 2. The FEV_1_ declines after placebo and after β_2_-agonist lead to an absolute effect of 41 percentage point (pp) less decline in FEV_1_ (based on 70–29), and to a relative effect of 58% less decline after the β_2_-agonist (based on 41/70). If there was no absolute mean effect, this leads to a 0% relative effect (0/70 = 0), and if all FEV_1_ decline was fully prevented, this leads to a 100% effect (70/70 = 1)

The absolute effect of a β_2_-agonist for a single participant was measured as the percentage point (pp) difference in the maximal exercise-induced FEV_1_ percent decline after β_2_-agonist treatment minus the maximal exercise-induced FEV_1_ percent decline after placebo treatment, see Fig. 1 as an illustration.

At the individual level, the relative effect was measured as the percent of the exercise-induced FEV_1_ percent decline prevented by β_2_-agonist treatment. It is calculated as the absolute effect divided by the maximal exercise-induced FEV_1_ percent decline after placebo (Fig. 1).

The usefulness of the absolute scale (intercept) and the relative scale (slope) in explaining the variation in β_2_-agonist effects on FEV_1_ decline after exercise was analyzed with linear mixed-effects models. The type of β_2_-agonist and the identity of the trial were used as clustering variables for the participants. The *lmer* function of the *lme4* package of the statistical software R was used for the mixed-effects modeling [33, 34].

First, the intercept, corresponding to the mean effect of a β_2_-agonist on the absolute scale, was included in the statistical model of the IPD, see formula (1) below. Thereafter, the slope was added, which explains the variation in the β_2_-agonist absolute effects on FEV_1_ decline by the variation in FEV_1_ declines after placebo administration (i.e. untreated FEV_1_ decline), formula (2) below. Finally, the intercept was removed, so that the slope remaining alone describes the mean effect of β_2_-agonists on the relative scale, formula (3) below. The models were compared with the anova test and Akaike Information Criterion (AIC). For the printouts of the calculations, see Additional file 1.

Definitions (see Fig. 1):

X = FEV_1_ decline in the placebo test,

Y = absolute difference in FEV_1_ declines in the β_2_-agonist and placebo tests

1 $$ {\mathrm{Y}}_{\mathrm{i}}=\upalpha +{\upvarepsilon}_{\mathrm{i}} $$

2 $$ {\mathrm{Y}}_{\mathrm{i}}=\upalpha +\upbeta \cdotp {\mathrm{X}}_{\mathrm{i}}+{\upvarepsilon}_{\mathrm{i}} $$

3 $$ {\mathrm{Y}}_{\mathrm{i}}=\upbeta \cdotp {\mathrm{X}}_{\mathrm{i}}+{\upvarepsilon}_{\mathrm{i}} $$

The median and interquartile levels for the relative effects of β_2_-agonists were calculated with the *rq* function of the *quantreg* package in R without adding an intercept [33, 35].

The study-level mixed-effects models were carried out with the *lmer* function with the type of β_2_-agonist being the clustering variable for studies and by using the number of participants as the weight for the study means. Similarly, as in the analysis of the IPD data, first the intercept was included, then the slope was added, and finally the intercept was removed.

Standard meta-analysis comparing the relative and absolute scales was performed using the generic inverse variance and random effects options of the RevMan program [3]. The meta-analyses were restricted to the 14 trials with the IPD, since the absolute mean effect on the FEV_1_ decline and the standard error (SE) for the difference could be calculated accurately from the individual paired differences from the IPD.

For the standard meta-analysis, the relative effects of individual trials were calculated in two ways, see Additional file 1: Table S3. First, the absolute mean effect and its SE (see above) were divided by the exercise-induced FEV_1_ decline after the placebo. If there was no absolute mean effect, this leads to a 0% relative effect; and if all FEV_1_ decline was fully prevented, this leads to a 100% effect. Second, the relative effect and its SE were derived from the slopes of the linear regression models of the 14 trials, see Additional file 1. We used the χ^2^ test and the *I*^2^ statistic to assess statistical heterogeneity among the trials in each meta-analysis [36]. A value of *I*^2^ greater than about 70% indicates a high level of heterogeneity.

**Fig. 2:**
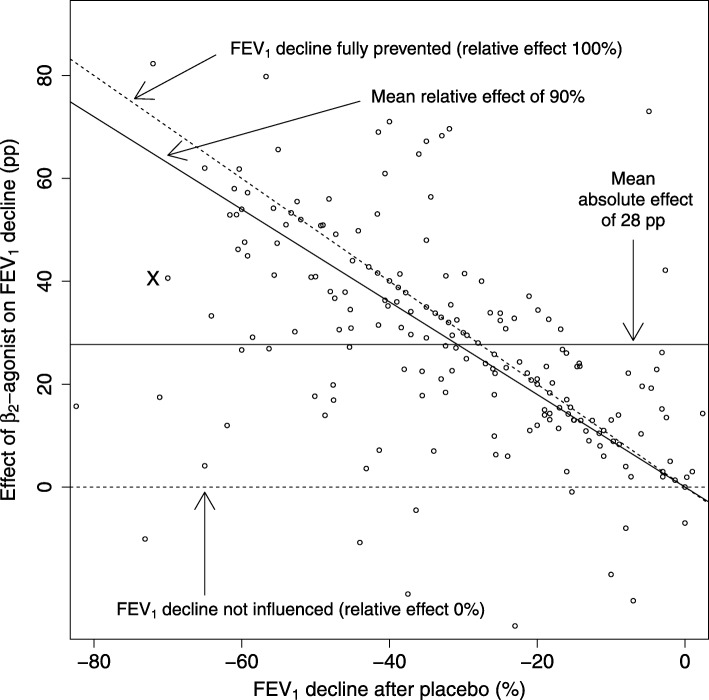
The effect of β_2_-agonists on exercise-induced bronchoconstriction in the IPD analysis. Each dot indicates one participant. The exercise-induced FEV_1_ decline after placebo is shown on the horizontal axis. The absolute effect of a β_2_-agonist is defined as the percentage point (pp) difference in the FEV_1_ declines after placebo and after β_2_-agonist, and is shown on the vertical axis, compare with Fig. 1. Lack of any effect of β_2_-agonist is indicated by the horizontal dashed line at the level of 0 pp., corresponding also to the relative effect of 0% (i.e. the FEV_1_ decline after β_2_-agonist is identical to the FEV_1_ decline after placebo treatment). Full removal of exercise-induced FEV_1_ decline is indicated by the upper diagonal dashed line corresponding to the relative effect of 100% (i.e. the FEV_1_ decline after placebo treatment is fully reversed after β_2_-agonist treatment). The mean absolute effect, defined by the intercept alone, is shown by the horizontal solid line at the level of 28 pp. The mean relative effect, defined by the slope alone, is shown by the diagonal solid line at the level of 90%. Abbreviations: pp., percentage points

To estimate the potential role of the regression to the mean phenomenon as a cause for the slope between the effect of β_2_-agonists and the placebo-test FEV_1_ decline in Fig. 2, we used three approaches. The slope generated by regression to the mean depends on the within-subject SD of the placebo-test FEV_1_ decline and the β_2_-agonist-test FEV_1_ decline, and on the between-subject SD of the placebo-test FEV_1_ decline [37]. Thus, regression to mean is independent of the size of treatment effect. Therefore, we first estimated the slope generated by regression to the mean by comparing two different placebo-test FEV_1_ declines of 45 participants of four studies [20, 23, 24, 30]. Second, we used the Blomqvist formula to calculate the corrected slope [37]. Third, we calculated from formula (1) in Hayes paper [37] the magnitude of within-subject SD that would be needed to generate the observed slope by the regression to mean phenomenon.

Two-tailed *P*-values were used.

## Results

### Analyses of the IPD

Fourteen placebo-controlled cross-over comparisons were identified that reported the IPD of the effects of β_2_-agonists on exercise-induced FEV_1_ decline (Table 1). In all they included 187 participants. Six trials with 89 participants used salbutamol (albuterol) [17–22], 3 trials with 36 participants used salmeterol [23–25], 2 trials with 24 participants terbutaline [26, 27], 1 trial with 16 participants reproterol [28], 1 trial with 12 participants bitolterol [29] and 1 trial with 10 participants used metaproterenol (orciprenaline) [30].

The included trials administered placebo and β_2_-agonist to the same participants in a cross-over design. The exercise challenge was carried out thereafter and the post-exercise decline in FEV_1_ was measured. Figure 1 demonstrates the calculation of the absolute effect of β_2_-agonist for participant X, who is also indicated in Fig. 2.

The level of exercise-induced FEV_1_ decline in the placebo test is shown on the horizontal axis of Fig. 2 for each individual participant in the 14 IPD studies. After placebo, the FEV_1_ changes caused by exercise ranged from an 82% decrease to a 2% increase, with the median of a 31% decrease. The absolute effect of the β_2_-agonist for each individual was calculated as the percentage point (pp) difference in the FEV_1_ decline after the β_2_-agonist and after the placebo. For example, participant X on the left-hand side of Fig. 2 had an exercise-induced FEV_1_ decline of 70% after placebo, and a decline of 29% after salbutamol, which indicates a 41 percentage point (pp) improvement (based on 70–29), as the effect of salbutamol on the absolute scale, see also Fig. 1. On the relative scale, the FEV_1_ decline of the same person was reduced by 58%, based on the ratio of 41/70.

On the relative scale, the 0% effect indicates that the β_2_-agonist has no effect, i.e., the FEV_1_ decline after β_2_-agonist is identical to the FEV_1_ decline after placebo. The 100% effect indicates full protection so that exercise after β_2_-agonist causes no decline in FEV_1_, i.e., the FEV_1_ decline occurring after placebo is fully reversed by the β_2_-agonist. These two limits are shown in Fig. 2 by the dashed lines. Ten participants showed β_2_-agonist effects below 0% which means that exercise-induced FEV_1_ decline in the β_2_-agonist test was greater than in the placebo test. Probably this is explained by random variation. Sixty-four participants showed β_2_-agonist effects over 100% which means that FEV_1_ level after exercise in the β_2_-agonist test was greater than the FEV_1_ level before exercise. In addition to random variation, this finding is also explained by our usage of the pre-drug level as the baseline. For many participants β_2_-agonist increased pre-exercise FEV_1_ level and if exercise-induced FEV_1_ decline is simultaneously prevented, this would lead to effects above 100% in the calculation of the relative effects.

The distribution of the data points in Fig. 2 suggests that the absolute effect of β_2_-agonist on FEV_1_ decline appears to be greater in study participants with larger baseline exercise-induced FEV_1_ declines after placebo on the left-hand side of the graph. This is tested explicitly below by initially fitting the data using only a single intercept which is equivalent to describing the effect of β_2_-agonist on exercise-induced FEV_1_ decline as a single average percentage point improvement akin to an absolute scale approach used by Bonini et al. [11]. Thereafter we fit the data using a slope to derive an average proportion of exercise-induced FEV_1_ decline that is reversed by β_2_-agonist treatment which accounts for differing baseline exercise-induced FEV_1_ declines akin to a relative scale approach.

The usefulness of the absolute scale (intercept) and the relative scale (slope) were compared with linear mixed-effects models shown in Table 2. When only the intercept was included, the β_2_-agonists reduced the FEV_1_ decline by an average of 28 pp. This intercept indicates the mean effect on the absolute scale. When the slope was added, the fit of the model was improved substantially. Furthermore, addition of the slope caused the estimate of the intercept to decrease substantially. When the intercept was removed from the model, the change in the fit of the model was much smaller compared with the addition of the slope. In addition, the lower AIC value also indicates that the model with the slope alone is better than the model with the intercept alone (Table 2). The slope of − 0.90 indicates that β_2_-agonist treatment reduced the exercise-induced FEV_1_ decline by 90% on the relative scale. The absolute effect (intercept alone) and the relative effect (slope alone) obtained from the mixed-effects models are shown in Fig. 2 as continuous solid lines.

**Table 2:** Comparison of the intercept and slope to explain the effect of β_2_-agonists by IPD

	Intercept	Slope	χ^2^(3 df) *	*P* *	AIC **
Uniform effect (absolute scale)	27.7	–			1638.3
Intercept and slope	7.9	−0.69	82.3	10^−17^	
Slope (relative scale)	–	−0.90	13.9	0.003	1576.8

The intercept and the slope were calculated with a linear mixed-effects model using the type of β_2_-agonist and study as grouping variables. The intercept alone indicates that β_2_-agonists decrease the post-exercise FEV_1_ decline by an absolute effect of 27.7 pp. (95% CI: 22.1 to 33.4 pp). The slope alone indicates that β_2_-agonists decrease the post-exercise FEV_1_ decline by a relative effect of 90% (95% CI: 72% to 109%). Abbreviations: pp., percentage points

* Anova test comparing the model on the row with the model on the row above

** AIC, Akaike information criterion. AIC estimates the relative quality of statistical models, lower AIC value is preferable

A further measure to compare the relative scale (slope) and the absolute scale (intercept) was the magnitude of the residuals of the models. For the absolute effect of the uniform 28 pp. decrease in FEV_1_ decline, the median residual was 10.8 pp. For the relative effect of the 90% decrease in FEV_1_ decline, the median residual was just 5.8 pp. This also illustrates that the relative effect (slope) captures much better the individual-level variation in the effects of β_2_-agonists than the absolute effect (intercept).

Variation in the effects of the β_2_-agonists was also analyzed by stratifying participants to categories by the post-exercise FEV_1_ declines after placebo administration. Over the 5 categories shown in Table 3, there is a 3-fold variation in the absolute mean effect of the β_2_-agonists between the extremes ranging from 15.2 pp. in the category with the lowest FEV_1_ decline after placebo administration to 44.3 pp. in the category with the highest FEV_1_ decline after placebo administration. The confidence intervals of the first, fourth and fifth groups are inconsistent with the overall mean absolute effect of 28 pp. decrease in FEV_1_ decline (Fig. 2). However, the confidence intervals of the relative effects of all the 5 categories are overlapping and consistent with the confidence interval of the overall 90% mean effect calculated from the slope of the linear regression model (Fig. 2, Table 2).

**Table 3:** Analysis of β_2_-agonist effects on FEV_1_ decline in categories of placebo-test FEV_1_ declines

FEV_1_ decline after placebo (range)	Mean FEV_1_ decline after placebo	Absolute effect on FEV_1_ decline (pp) (95% CI)	Relative effect (95% CI)	N
10–19%	15.5%	15.2 (10.2–20.3)	0.99 (0.79–1.24)	33
20–29%	24.7%	23.6 (17.7–29.9)	0.92 (0.74–1.14)	29
30–39%	34.5%	33.0 (25.0–40.9)	0.95 (0.78–1.15)	34
40–49%	44.5%	39.7 (30.6–49.3)	0.83 (0.69–1.00)	29
50–83%	59.9%	44.3 (34.8–54.6)	0.71 (0.60–0.84)	34

Footnote: this table is restricted to 159 participants who had exercise-induced FEV_1_ decline of ≥10% in the placebo test. The 95% CI for the relative effect was calculated with the approach described in [5], see Additional file 1. Abbreviations: pp., percentage points

**Fig. 3:**
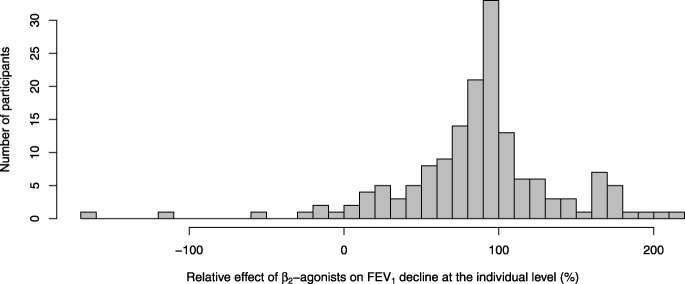
Distribution of the individual-level relative effects of β_2_-agonists on exercise-induced FEV_1_ declines. This figure is limited to 159 participants who had ≥10% decline in FEV_1_ by exercise in the placebo test. Participants with small FEV_1_ declines are not informative in the calculation of participant-level relative effect. In addition, the 10% limit is usual for the diagnosis of exercise-induced bronchoconstriction [12]. Participant X of Figs. 1 and 2 has value 0.58 (58%) in this figure. As in Fig. 2, some participants showed β_2_-agonist effects below 0% or over 100%

The distribution of the individual-level relative effect of β_2_-agonists was skewed with skewness of − 1.05 (Fig. 3). Therefore, the median effect might be a more informative descriptive measure of typical effect than the mean. The median relative effect over the 187 participants was an 88% reduction in FEV_1_ decline, with the interquartile range from 60% to 103%. Although the median is close to the mean estimate (90%), the asymmetry is apparent in the interquartile range.

### Estimation of the possible role of the regression to the mean

Regression to the mean is a potential confounder in the analysis of change by baseline values [37]. We used three approaches to evaluate the possible bias in the slope in Fig. 2 caused by regression to the mean.

First, four studies with a total of 45 participants carried out two separate placebo-exercise tests and they can be used to estimate the size of slope caused by regression to the mean when there is no treatment effect, and a slope of − 0.153 was observed, which is substantially smaller than the slope of − 0.691 for the model with slope and intercept (Table 2).

Second, the within-subject SD for the placebo-test FEV_1_ decline from the four studies was 6.23 pp. and the observed between-subject SD for all 187 participants was 18.9 pp. The Blomqvist formula can be used to estimate the true slope from the previous SD values [37], and the estimated true slope in Fig. 2 is − 0.653, which is minimally different from our calculated slope of − 0.691 (95% CI: − 0.477 to − 0.910) for the model with slope and intercept (Table 2). In contrast, applying the Blomqvist formula to the slope of − 0.153 of the placebo-placebo comparison of the previous paragraph, the true slope becomes − 0.049 which is very close to the null slope as expected.

Third, we calculated that to generate a slope of − 0.69, the within-subject SD for the measurement of FEV_1_ decline should be up to 28 pp., which is over 4 times the observed within-subject SD (ie 6.23 pp).

Thus, on the basis of these three approaches, the size of the regression to the mean phenomenon is so small that it has no practical relevance in our analysis of the IPD in Fig. 2.

### Analysis of study-level data by the mixed-effects models

**Fig. 4:**
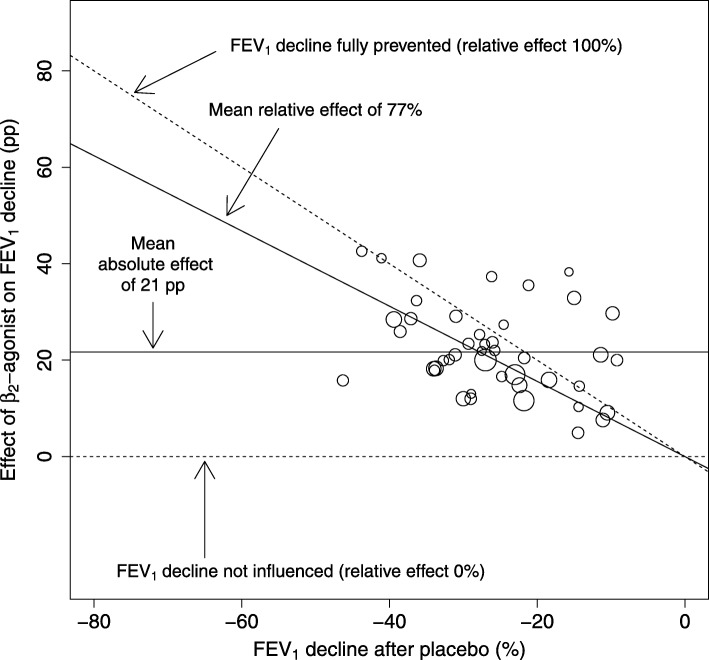
The effect of β_2_-agonists on exercise-induced bronchoconstriction in the study-level analysis. The studies in this analysis are the same as those in the Analysis 1.1 of Bonini et al. [11]. Each circle indicates one study and the area of the circle is proportional to the number of participants and represents the weighting of the study in calculating the mean absolute and relative effects. See Fig. 2 for the description of the lines in this figure. The mean absolute effect, defined by the intercept alone, is shown by the horizontal solid line at the level of 21 pp. The mean relative effect, defined by the slope alone, is shown by the diagonal solid line at the level of 77%. For the identity of the 44 trials and their references, see Additional file 1: Table S2 and [11]. Abbreviations: pp., percentage points

The study-level mixed-effects model was focused on the 44 cross-over trials [11] which reported the mean exercise-induced FEV_1_ decline after β_2_-agonist and after placebo (Fig. 4). The study mean FEV_1_ declines after the placebo ranged from − 46% to − 9% with a median decline of − 27%. The range of the data points is much narrower compared with the range of the IPD (Fig. 2), resulting in the study-level analysis having less statistical power to compare the absolute and relative scales. Unlike the data points in Fig. 2, given the narrower range of FEV_1_ declines after placebo along the x-axis in Fig. 4, it is less clear whether the effect of β_2_-agonist on FEV_1_ decline is greater in studies with larger mean baseline exercise-induced FEV_1_ declines after placebo.

Testing this formally, the intercept of the study-level analysis indicated a mean uniform 21 pp. reduction in exercise-induced FEV_1_ decline by β_2_-agonists (Fig. 4). The slope indicates a 77% reduction in exercise-induced FEV_1_ decline by β_2_-agonists. Analysis of the study-level data suggests that the intercept might be more consistent with the data (Table 4).

**Table 4:** Comparison of the intercept and slope to explain the effect of β_2_-agonists by study means

	Intercept	Slope	χ^2^(3 df) *	*P* *	AIC **
Uniform effect (absolute scale)	21.4	–			323.5
Intercept and slope	16.4	−0.24	8.52	0.036	
Slope (relative scale)	–	−0.77	11.9	0.010	333.0

The intercept and the slope were calculated with a linear mixed-effects model using the type of β_2_-agonist as the grouping variable. The studies were weighted by the square root of the number of participants. For the identity of the 44 trials and their references, see Additional file 1: Table S2 and [11]. Abbreviations: pp., percentage points

* Anova test comparing the model to the model in the row above

** AIC, Akaike information criterion. AIC estimates the relative quality of statistical models, lower AIC value is preferable

### Standard meta-analysis of study-level data

Standard meta-analyses comparing the absolute and two relative scales were limited to the 14 trials which reported IPD as these allowed calculation of individual paired differences and their SE values. The calculation of the 95% CIs by the absolute scale and the two relative scales is illustrated in Additional file 1: Table S3.

**Fig. 5:**
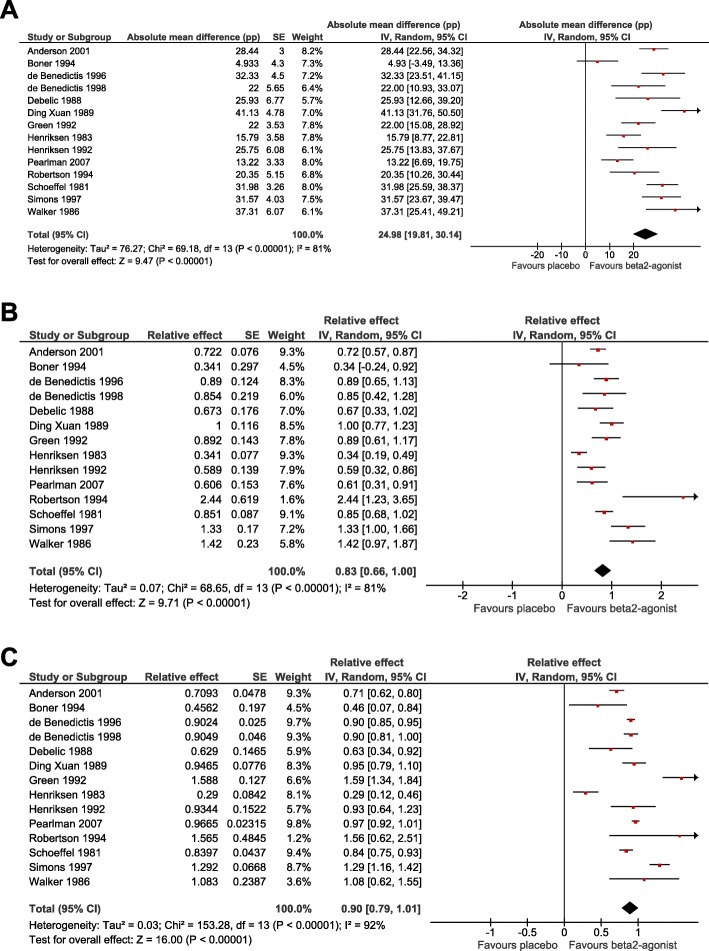
Meta-analysis of the effect of β_2_-agonists on exercise-induced FEV_1_ declines **a**) on the absolute scale, **b**) on the relative scale based on the absolute scale, and **c**) on the relative scale based on the slopes of linear regression models. See Additional file 1: Table S3 for the description of the calculation of the 95% CIs. In the forest plot on the right-hand side, the vertical line indicates the placebo test level. The horizontal lines indicate the 95% CI for the β_2_-agonist effect in the particular trials and the square in the middle of the horizontal lines indicate the point estimate of the effect in the trial. The sizes of the squares indicate the relative weights of the trials in the pooling of the results. The diamond shape indicates the pooled effect and its 95% CI. The generic inverse variance and random effects options of the RevMan program were used [3]. See Additional file 1 for the details of the calculations

There is substantial heterogeneity between the trials on the absolute scale with *I*^*2*^ = 81% (Fig. 5a). The estimate of a uniform 25 pp. mean reduction is similar to the absolute scale estimate from the mixed-effects model using IPD (Table 2).

The study-level relative effects were first obtained by dividing the absolute effect by the exercise-induced FEV_1_ decline in the placebo test for each study prior to pooling, see Additional file 1: Table S3 for the explanation of this transformation. In this approach the heterogeneity between the studies was also *I*^*2*^ = 81% (Fig. 5b). This approach indicates that β_2_-agonists reduced exercise-induced FEV_1_ decline by 83%. This estimate is similar to the mean effect on the relative scale in the mixed-effects model using IPD (Table 2). There is no substantial difference in the statistical significance between the relative effect calculated in this way (Z = 9.7: Fig. 5b) compared with the pooled absolute effect (Z = 9.5; Fig. 5a).

The relative effects of the IPD studies were also obtained from the slopes of linear regression models similar to the analysis presented in Fig. 2, however, now for each of the individual trials separately (Fig. 5c). For most studies, this approach led to smaller SE values compared to the Fig. 5b analysis. The smaller SE values increased the heterogeneity between the studies to *I*^*2*^ = 92%, yet concurrently increased the precision of the pooled estimate substantially (Z = 16.0; Fig. 5c), compared with Fig. 5a and b. This approach indicates that β_2_-agonists reduced FEV_1_ decline by 90%, identical to the slope estimate calculated in Table 2.

## Discussion

The goal of this study was to compare whether the absolute or the relative scale yields more consistent estimates of effect, using the example of β_2_-agonist treatment to prevent FEV_1_ declines associated with EIB, the severity of which can range widely between patients. The absolute scale is routinely used in the analysis of continuous data and therefore the comparison of these two scales is relevant more widely than just for the analysis of FEV_1_ changes.

In people with EIB, Bonini et al. calculated that the β_2_-agonists decreased exercise-induced FEV_1_ decline by 17.67 pp. (95% CI: 15.84 to 19.51 pp) [11]. If EIB was a homogeneous medical condition, such a uniform effect might be meaningful. Instead, EIB is highly heterogeneous, since it is usually defined by post-exercise FEV_1_ decline of 10% or more, though other arbitrary cut-off limits have been used. Thus, in this dichotomization two persons with 11% and 80% FEV_1_ declines after exercise are both classified as having EIB, whereas a person with a 9% FEV_1_ decline is not. However, the person who has the 11% decline probably is biologically much closer to the person who has the 9% decline compared with the person who has the 80% FEV_1_ decline after exercise. It does not seem reasonable to assume that Bonini’s estimate of 17.67 pp. effect would apply for people with a low and a high level of exercise-induced FEV_1_ decline. Furthermore, dichotomization of continuous variables decreases statistical power [38–41].

One approach to achieve more personalized effects of β_2_-agonists is to categorize people into groups by their untreated exercise-induced FEV_1_ decline levels (Table 3). In people who had untreated exercise-induced FEV_1_ declines in the range from 10% to 19%, β_2_-agonists reduced the FEV_1_ decline by 15 pp. (95% CI: 10 to 20 pp), whereas in people who had untreated FEV_1_ declines in the range from 30% to 39%, the reduction of the decline was 33 pp. (95% CI: 25 to 41 pp), and in people who had untreated FEV_1_ declines of 40% and greater the percentage point improvement was even greater (Table 3). The confidence intervals of the three groups with FEV_1_ decrease 30% and greater are all inconsistent with the 17.67 pp. effect calculated by Bonini [11]. These three groups contain 61% (97 of 159) of the participants in Table 3. This illustrates that Bonini’s estimate of effect does not apply to a great proportion of people classified as having EIB.

The relative scale is most informative in the analysis of the β_2_-agonist effects on exercise-induced FEV_1_ declines since on the relative scale a single estimate of effect, expressed as a percentage improvement of the baseline exercise-induced FEV_1_ decline (rather than a uniform percentage point improvement), applies over all study participants independent of their initial FEV_1_ decline levels (Fig. 2, Tables 2 and 3). In our analysis, half of the participants with IPD had observed β_2_-agonist effect 5.8 pp. or more distant from the mean 90% effect, which also shows that the relative scale better captured the observed β_2_-agonist effect compared with the use of a single uniform 28 percentage point improvement, which had median residual of 10.8 pp.

In our study, the primary comparison of the absolute and the relative scales was based on IPD, since the wide distribution of FEV_1_ declines in the IPD analysis results in greater statistical power to compare intercepts and slopes. We also compared the absolute and relative scales on the basis of study-level data of 44 trials, but no superiority of the relative scale was seen in that comparison, indeed absolute scale seemed to be slightly better (Table 4). In addition, no superiority of relative scale over the absolute scale was seen in standard meta-analyses (Fig. 5a and b). These discrepancies between the analyses based on IPD (Fig. 2) and on the study-level data are examples of the “ecological fallacy”. In order to avoid the potential for the ecological fallacy introduced by study-level analyses, whenever feasible, examination of IPD has been recommended [42–44]. Thus, analysis of the study-level data alone (Table 4) or the comparison of standard meta-analyses (Fig. 5a and b) would have led to a false conclusion that the absolute scale is better or at least not worse than the relative scale.

Nevertheless, even though the analyses of the study-level data did not yield valid comparison of the absolute and relative scales, the study-level estimate calculated from 44 trials for the relative effect was quite similar with the estimate from the IPD analysis of 14 trials: 77% vs. 90% improvement in the exercise-induced FEV_1_ decline, respectively. This divergence in estimates can be partly explained by the different sets of studies that were compared. The standard study-level meta-analyses of the 14 studies which had IPD available reached relative effect estimates of 83% and 90% reduction in FEV_1_ decline, depending on the calculation of the SE (Fig. 5), very similar to the overall IPD mixed-effects regression analysis. This latter comparison was based on the same set of studies.

Most popular statistical software such as the RevMan of the Cochrane Collaboration do not have an option to pool continuous outcomes on the relative scale. However, it is available in the *metacont* function of the R package *meta* [33, 45, 46]. Nevertheless, a simple approach to pool results of study-level data on the relative scale when this option is not available in a statistical program is to normalize the results of the studies by dividing the absolute mean effects and their SD values by the placebo group mean outcome value (Table S3). Such a transformation can easily be done with a spreadsheet program and the transformed data can be entered in a standard statistical program for meta-analysis. This approach of calculating the relative effect is illustrated in Fig. 5b. Alternatively, if IPD is available, one can calculate and pool the slopes of linear regression curves for each study, which usually leads to more narrow SE estimates and more accurate pooled estimates as shown in Fig. 5c. However, IPD is rarely available and therefore calculation of the slope is not often feasible. Furthermore, for many cross-over trials that reported the study-level data (Fig. 4), the paired SE was not published and would need to be imputed, but this problem applies to both the absolute and the relative scales.

In meta-analysis of binary outcomes, relative scale analysis using effect measures such as risk ratios or odds ratios leads to asymmetric confidence intervals, because the studies are pooled on the logarithmic scale with symmetric confidence intervals and then transformed back. Similarly, in meta-analysis of continuous outcomes, the findings can be pooled on the logarithmic scale using ratio effect measures, leading to asymmetric CIs [5]. However, relative scale effects for continuous outcomes can also be derived from slopes (Fig. 2), or by the normalization of the results of the studies by dividing the absolute mean effects and their SD values by the placebo group mean outcome value (Table S3) [10], both of which lead to symmetric CIs on the relative scale. Therefore, CIs of the continuous outcomes are not necessarily asymmetric.

The distribution of the relative effects at the individual level is skewed (Fig. 3). Therefore, the median relative effect might appear a more useful descriptive estimate than the mean relative effect. Study-level meta-analyses cannot find the median effect nor can they describe the distribution of the individual-level effects such as the interquartile range. Thus, the IPD analysis can give important information additional to the study-level analyses. In our case, the difference between the mean effect of 90% and the median effect of 88% prevention of EIB is minor. Nevertheless, the great variation in the individual-level effects indicates that the efficacy of a particular β_2_-agonist in protecting against EIB needs to be assessed at the individual level (Fig. 3).

This study was motivated by Bonini’s meta-analysis on β_2_-agonists for exercise-induced FEV_1_ declines and their use of the absolute scale in the analysis of study results [11]. However, the absolute scale, either as percentage point differences or as volume differences (measured in Liters), has been used in the analysis of FEV_1_ changes in several other meta-analyses of the Cochrane Library [47–53]. Thus, the superiority of the relative scale is not just an issue relevant to Bonini’s meta-analysis. For example, one of the Cochrane reviews [53] estimated the effect of vitamin C on EIB on the absolute scale and described the effect of vitamin C five minutes after exercise in the Schachter (1982) trial [54] as follows: “No significant difference between vitamin C and placebo: Vitamin C mean: –0.24 (SE ± 0.06) L/s, Placebo mean: –0.44 (SE ± 0.14) L/s, t = 2.13 (P = 0.057)” [53]: Table 2. However, the slope of a linear regression analysis of the Schachter study [54], which had reported the IPD, indicated that vitamin C’s relative decrease in FEV_1_ decline was highly significant: 55% (95% CI: 32 to 78%; *P* = 0.0003) [55]. This difference in *P*-values also illustrates that the calculation of the absolute effect, which is the custom in the Cochrane reviews, can lead to false negative conclusions.

Our study did not intend to reproduce Bonini’s main meta-analysis, which was labeled Analysis 1.1 in their paper [11]. There were several errors and data extraction inconsistencies, some of which were severe, see Additional file 1: Table S4. We used Bonini’s review as an example to demonstrate that the calculation of absolute effects can lead to suboptimal effect estimates. Similar to Bonini’s analysis, we combined different β_2_-agonists to calculate one single estimate of effect. We took this approach because our primary goal was to compare two different methods in the analysis of FEV_1_ changes rather than estimating the effectiveness of a particular β_2_-agonist, or a particular experimental protocol for conducting an exercise test. If one β_2_-agonist or protocol is less effective than another, the lower effectiveness would be analyzed in both ways and, thereby, would contribute equally to both the relative and absolute scale analysis. We tried to reduce the heterogeneity of comparisons by selecting salbutamol (or if not tested, salmeterol) when several β_2_-agonists were investigated in the same report, the shortest delay between β_2_-agonist administration and exercise test when exercise tests were repeated several times after the administration of a β_2_-agonist, and pre-drug FEV_1_ as baseline when possible. Furthermore, we took into account the variations in β_2_-agonists and the conduct of exercise tests used among different trials by using the β_2_-agonist and the trial as clustering variables in the analyses.

Friedrich et al. compared the relative and absolute scales for diverse continuous outcomes and showed that, on average, the relative scale led to lower heterogeneity compared with the absolute scale indicating that the former is more informative [5–7]. In addition, previous analyses demonstrated that the analysis of effects on the duration of diseases and comparable outcomes is more informative on the relative scale than on the absolute scale [8–10]. However, there are many different kinds of contexts where continuous outcomes are generated and, therefore, the relative scale is not always applicable. Apparently, one requirement for using the relative scale is that there is a relevant 0% to 100% scale for the measurement. Such requirements are not always satisfied. For example, there are no reasonable 0% target levels for body weight, body temperature or blood pressure. In such cases, the relative scale may not be ideal.

Since in many contexts the relative scale is more informative in the analysis of continuous outcomes, the option to use the relative scale should be made widely available in meta-analysis software so that researchers can compare and decide themselves which scale is most suitable for their particular outcome.

## Conclusions

Compared with the absolute scale, the relative scale captures more effectively the variation in the effects of β_2_-agonists on exercise-induced FEV_1_ declines. The absolute scale has been widely used in the analysis of FEV_1_ changes and it may have led to sub-optimal statistical analysis in some cases. The choice between the absolute scale and the relative scale should be determined on the basis of biological reasoning and empirical testing to identify the scale that leads to lower heterogeneity. The relative scale option should be made available for meta-analysis software. Meanwhile the transformation to the relative scale can be easily calculated with spreadsheet programs and the transformed data can be analyzed with standard meta-analysis software.

## Supplementary information

**Additional file 1.** Details of the extraction of the IPD and study-level data. Some errors and inaccuracies in the Bonini et al. [11] data extraction are described. Description of the calculations.

**Additional file 2.** The data sets used in this analysis, calculation of the 95% CIs for the ratios in Table 3, measurements from published figures to yield numerical extracted FEV_1_ values.

## Data Availability

Data analyzed in this study are available in Additional file 2.

## Abbreviations

AIC: Akaike Information Criterion
IPD: Individual patient data
MD: Mean difference
RR: Relative risk
SD: Standard deviation
SE: Standard error
