# Supplementary material for: Many continuous variables should be analyzed using the relative scale: a case study of β2-agonists for preventing exercise-induced bronchoconstriction
Source: Syst Rev. 2019 Nov 19;8:282. doi: 10.1186/s13643-019-1183-5 (PMC6865024; doi:10.1186/s13643-019-1183-5)
Supplement: Supplementary file 1 — Additional file 1. Details of the extraction of the IPD and study-level data. Some errors and inaccuracies in the Bonini et al. [11] data extraction are described. Description of the calculations. [file 13643_2019_1183_MOESM1_ESM.pdf]

Many continuous variables should be analyzed using the relative scale: a case study of β_2-agonists for preventing exercise-induced bronchoconstriction

Harri Hemilä and Jan O. Friedrich

Supplementary File 1

This is supplementary material to a paper by Hemilä and Friedrich (2019)

Published in **Systematic Reviews**
https://systematicreviewsjournal.biomedcentral.com/

2019-8-7

Harri Hemilä
Department of Public Health,
University of Helsinki,
Helsinki, FIN-00014 Finland
harri.hemila@helsinki.fi
http://www.mv.helsinki.fi/home/hemila

**Contents**	**Page**
Explanations and Abbreviations:	2
Table S1: Extraction of IPD data of the 14 studies	3
Measurements of IPD findings of two studies from figures	4
Table S2: Extraction of the study means data	6
Table S3: Calculation of the absolute and relative effects for Fig. 5	10
Data extraction inconsistencies and errors in Bonini et al. (2013)	11
Table S4: Data extraction inconsistencies and errors in Bonini et al. (2013)	12
Printouts of statistical calculations	16

Explanations and Abbreviations:

Albuterol: a synonym in the USA for salbutamol

FEV_1: forced expiratory volume in 1 second (the volume a person is able to exhale in 1 s)

IPD: individual participant data

MDI: metered dose inhaler

“1 hour test” indicates exercise test carried out 1 hour after the drug administration

“Pre-drug as baseline” indicates that exercise-induced FEV_1 decline is calculated from the FEV_1 level before drug administration

“Post-drug as baseline” indicates that exercise-induced FEV_1 decline is calculated from the FEV_1 level after drug administration

Extraction of IPD data of the 14 studies

The methods of 12 IPD studies were described by Bonini et al. (2013).
The methods of the two studies listed below were not described by Bonini et al.
Robertson (1994): 8 nonsmoking asthmatic men. They were all taking β_2-agonists and regular inhaled corticosteroids. Inhaled corticosteroids were continued during the study.
Double-blinded, cross-over study.

Schoeffel (1981): 10 participants (3 male, 7 female) with asthma. They were all taking β_2-agonists and some used inhaled corticosteroids.
Single-blind randomized study.

Table S1: Extraction of IPD data of the 14 studies

**Study**	**Dose of β_2-agonist; IPD extracted from; time of exercise test after the drug**
Anderson (2001)	Salbutamol 200 μg<br>Table 2 (p. 896): 30 min test<br>The mean of Diskus and pMDI was calculated as the outcome
Boner (1994)	Salbutamol 200 μg<br>Table 3 (p. 937): 3 hour test
de Benedictis (1996)	Salmeterol 50 μg<br>Table 2 (p. 2101): 1 hour test
de Benedictis (1998)	Salbutamol 200 μg<br>Table 2 (p. 354): 20 min test
Debelic (1988)	Reproterol 1 mg<br>Table 1 (p. 27): 15 min test
Dinh Xuan (1989)	Terbutaline 500 μg<br>Fig 1 (p. 509): 15 min test<br>Max percent decrease in FEV_1 within 60 min measured from Fig 1, see p. 3
Green (1992)	Salmeterol 50 μg<br>Table 1 (p. 1015): 1 hour test; Table 2 (p. 1016): Pre-drug – Post-drug changes
Henriksen (1983)	Terbutaline 32.5 μg<br>Table 1, Before budesonide administration (p. 995): 15 min test<br>The FEV_1 decline is calculated as absolute decline (Δ) from B-2 (Baseline-2)
Henriksen (1992)	Salbutamol 200 μg<br>Table III (p. 1179): 30 min test (Test 1) Pre-drug – Post-drug changes
Pearlman (2007)	Salbutamol (levalbuterol 90 μg)<br>Table 2 (p. 732): 30 min test<br>Pre-drug as baseline
Robertson (1994)	Salbutamol 200 μg<br>Table 1 (p. 1980): 30 min test; calculated as Pre-drug – Post-exercise difference
Schoeffel (1981)	Metaproterenol 1.5 mg<br>Table I (p. 274): 15 min test (Test 1)
Simons (1997)	Salmeterol 50 μg<br>Fig 2A, Day 1 morning (p. 658): 1 hour test<br>Results were measured from the figure, see p. 4 of this Supplement
Walker (1986)	Bitolterol 1.0 mg<br>Table I (p. 34): 45 min test; calculated as Pre-drug – Post-exercise difference

Measurements of IPD findings of two studies from figures

Measurement of Dinh Xuan (1989) results from Fig 1

Dinh Xuan reported the effect of terbutaline on post-exercise FEV_1 decline for 10 participants in a figure, see below. The lowest FEV_1 value after exercise was measured with a graphics program and the maximal FEV_1 decline was calculated. See **Supplementary file 2** for the measurements and calculations.

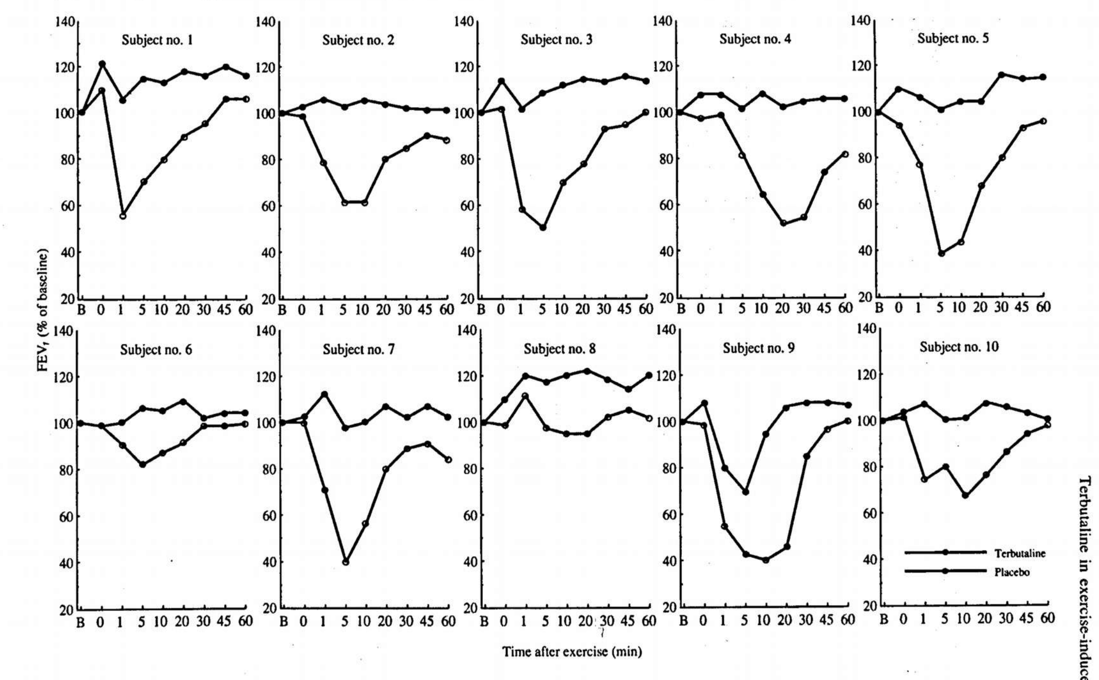

Fig. 1. Individual changes in forced expiratory volume in 1 s (FEV_1) from baseline (B) FEV_1 caused by exercise after treatment with 0.5 mg terbutaline or placebo by inhalation.

Measurement of Simons (1997) results from Fig 2A

Simons reported the FEV_1 levels (as % predicted) before treatment, and after treatment and exercise. Data for the same 14 participants are reported for both placebo (left) and salmeterol (right) tests, see the figure below. However, the lines overlap to such an extent that only 11 participants could be clearly identified for both the placebo and salmeterol tests. The 11 participants are indicated by the red lines and numbered from 1 to 11. See **Supplementary file 2** for the measurement and calculation of the FEV_1 changes in these 11 participants. Comparison of the mean and SD values we measured from the published figures and Simons report indicates close similarity in the means, see below. Thus, we were able to capture most of the findings.

	Simons Table 2 published	Our calculation Supplementary file 2
N:	14	11
Exercise-induced FEV_1 decline (%) mean±SD:		
Salmeterol		
Maximum fall	-7±6	-7.9±5.2
Placebo		
Maximum fall	24±12	23.6±11.4

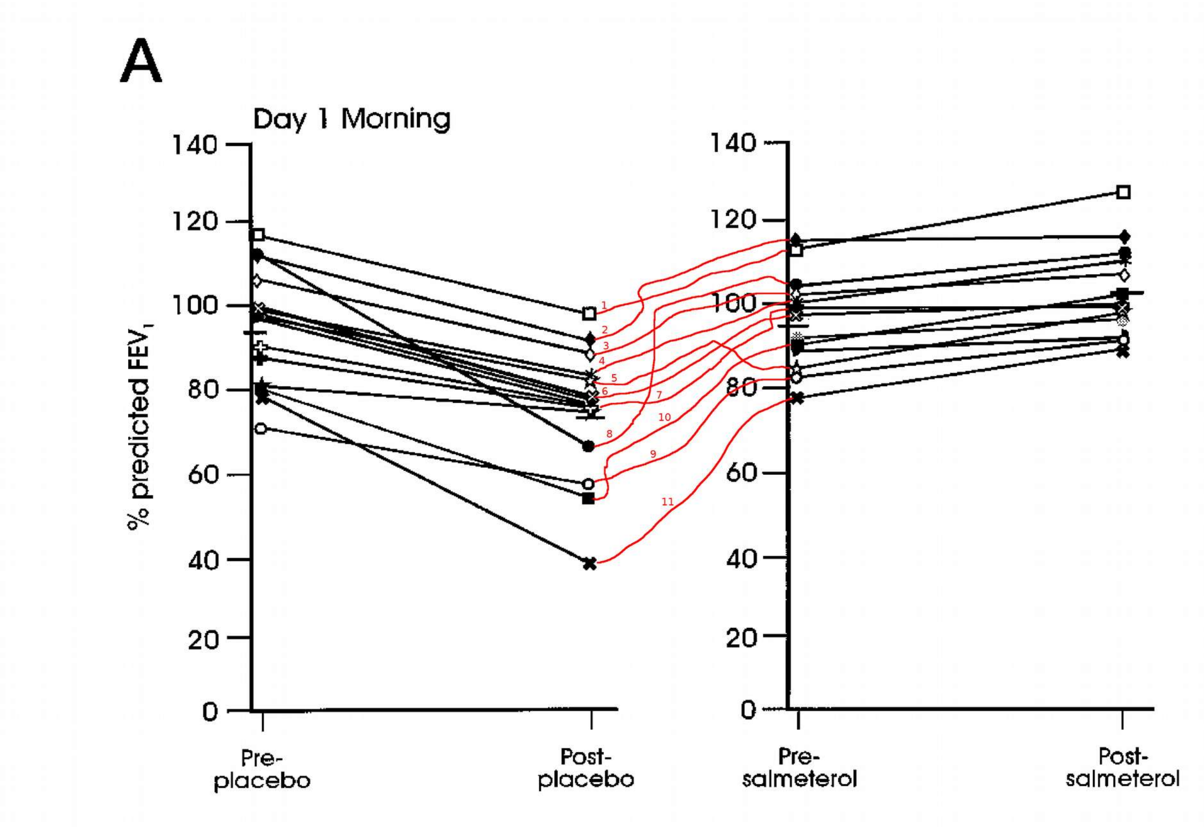

Extraction of the study means data

The following Table S2 describes the specific time points and the comparisons, from which we extracted the FEV_1 changes in the placebo and β_2-agonist tests.
The studies with IPD are listed to make this list consistent with Bonini's Analysis 1.1. but the IPD estimates are not added to this table, see Table S1.
Two parallel-group studies (Kemp 1994 and Vazquez 1984) are not included in our analysis.

For the references to the studies and to a description of the studies,
see Bonini [11]:
https://doi.org/10.1002/14651858.CD003564.pub3
https://www.ncbi.nlm.nih.gov/pubmed/24089311

The number of participants in the cross-over studies is indicated by N.

Table S2. Extraction of the study means data

Study	Dose of β_2-agonist;<br>IPD extracted from;<br>time of exercise test after the drug	N [a)]	FEV_1 change	
			β_2-Agonist	Placebo
Anderson (2001)	IPD	27		
Blake (1999)	Albuterol 180 µg<br>Table 3: 1 hour test<br>Pre-drug as the baseline	24	+9.7%	-11.4%
Boner (1994)	IPD	15		
Bronsky (1995)	Albuterol powder 200 µg<br>Table 1: 15 min test	44	-6%	-23%
Bronsky (1999)	Salmeterol Diskus 50 µg<br>Fig 1 and text: 1 hour test	24	-1.4%	-10.5%
Bronsky (2002)	Albuterol 180 µg<br>Fig 1: 15 min test	17	-8.5%	-37.1%
Carlsen (1995)	Salmeterol 50 µg<br>Table 2: 10-12 hour test	23	-18%	-30%
Cavagni (1993)	Salbutamol MDI 200 µg<br>Table 4: 10 min test	9	-15.92%	-28.93%
Clarke (1990)	Fenoterol 100 µg<br>Table 1, Day 2: 10 min test	20	+19.9%	-9.8%
Daughbjerg (1996)	Salbutamol 400 µg<br>Page 685 bottom: 3 hour test,<br>the "median" is reported, but we analyze it as an approximation to the mean.	15	-17%	-29%
Debelic (1988)	IPD	16		
De Benedictis (1996)	IPD	12		
De Benedictis (1998)	IPD	12		
Del Col (1993)	Albuterol MDI 200 µg<br>Table 3: 10 min test	15	-2.37%	-26.06%
Dinh Xuan (1989)	IPD	10		

Study	Dose of β_2-agonist;<br>IPD extracted from;<br>time of exercise test after the drug	N [a)]	FEV_1 change	
			β_2-Agonist	Placebo
Egglestone (1981)	Terbutaline 500 µg<br>Table I: 1 hour test<br>Comparison to the Pre-drug level<br>Calculations of the FEV_1 declines are as follows:<br>*Terbutaline:*<br>FEV_1 change of 0.37 L is 10% of the Pre-drug level, thus Pre-drug level is 3.7 L. Pre-drug to Post-drug is<br>+0.37 L and Post-drug to Post-exercise is -0.44 L; thus, Pre-drug to Post-exercise is -0.07 L. Thus FEV_1 decline is<br>-1.9% (= -0.07 L/3.7 L).<br>*Placebo:*<br>Pre-exercise FEV_1 level is<br>3.56 L (=1.14 /0.32). Pre-drug is<br>3.56 L – 0.05 L = 3.51 L. Thus, FEV_1 decline:<br>(-1.14 + 0.05)/3.51 = 31%	17	-1.9%	-31%
Ferrari (2000)	Formoterol 12 µg<br>Page 511 middle: 15 min test	14	-5.9%	-29.3%
Green (1992)	IPD	13		
Grönneröd (2000)	Formoterol 9 µg<br>Table 2: 15 min test	27	-2.5%	-18.4%
Hawksworth (2002)	Ventolin HFA 180 µg<br>Fig 1 and p. 475 left: 30 min test	24	-15.4%	-33.7%
Henriksen (1983)	IPD	14		
Henriksen (1992)	IPD	12		
Hills (1976)	Salbutamol: 200 µg<br>Fig 2: 20 min test<br>Initial vs. 5 min after exercise	19	+4.8%	-35.9%
König (1981)	Metaproterenol inhaler 1.3 mg<br>Table 3: 1 hour test (Study 2)<br>Pre-drug as the baseline level.<br>Calculations of the FEV_1 declines are as follows:<br>*Metaproterenol:*<br>FEV_1 was increased by 20% by metaproterenol (i.e. Post-drug level is 120%). Post-drug to Post-exercise decline is 19% of the 120%. Thus FEV1 decline is<br>-15.8% (= -19%/120%) from Pre-drug level.<br>*Placebo:*<br>FEV_1 was increased by 6% by placebo (i.e. Post-drug level is 106%). Post-drug to Post-exercise decline is 36% of the 106%. Thus FEV1 decline is<br>-34% (= -36%/106%) from the Pre-drug level.	24	-15.8%	-34%

Study	Dose of β_2-agonist; IPD extracted from; time of exercise test after the drug	N a)	FEV$_1$ change	
			β_2-Agonist	Placebo
König (1984)	Fenoterol 0.8 mg<br>Table 2: 10 min test (Run 1)	12	-2.5%	-27.8%
Larsson (1982)	Fenoterol 400 µg<br>Fig 1: 10 min test<br>Pre-drug as baseline	8	+22.6%	-15.7%
McAlpine (1990)	Salbutamol 200 µg<br>Table 1: 2 hour test<br>Comparison Pre-drug vs. 2 hour test<br>Calculations of the FEV$_1$ declines are as follows:<br>*Salbutamol:*<br>FEV$_1$ was increased by 10.0% by salbutamol (i.e. Post-drug level is 110% = 3.39 L/3.08 L).<br>Post-drug to Post-exercise decline is 14.1% of the 110%. Thus FEV$_1$ decline is -12.8% (= -14.1%/110%) from the Pre-drug level.<br>*Placebo:*<br>FEV$_1$ was not changed by placebo. Post-drug to Post-exercise decline is 32.7%, which is thus also Pre-drug to Post-exercise change.	12	-12.8%	-32.7%
McFadden (1986a)	Albuterol 200 µg<br>Table II: 15 min test<br>Comparison Pre-drug vs. 10 min<br>Calculations of the FEV1 declines are as follows:<br>*Albuterol:*<br>3.58/3.23 = 1.108 → +10.8%<br>*Placebo:*<br>2.95/3.25 = 0.908 → -9.2%	15	+10.8%	-9.2%
McFadden (1986b)	Albuterol 180 µg<br>Table II: 15 min test<br>Comparison Pre-drug vs. 5 min<br>Calculations of the FEV$_1$ declines are as follows:<br>*Albuterol:*<br>3.69/3.13 = 1.179 → +17.9%<br>*Placebo:*<br>2.67/3.14 = 0.850 → -15.0%	20	+17.9%	-15.0%
Morton (1989)	Rimiterol 400 µg<br>Fig 1 and p. 64 left top: 2 min test	10	+2.807%	-24.54%
Newnham (1993)	Salbutamol 200 µg	11	-3.8%	-27.1%

Study	Dose of β_2-agonist;<br>IPD extracted from;<br>time of exercise test after the drug	N [a)]	FEV$_1$ change	
			β_2-Agonist	Placebo
	Fig 1 and p. 441: 1 hour test<br>Pre-drug as the baseline<br>Calculations of the FEV$_1$ declines are as follows:<br>*Salbutamol:*<br>FEV$_1$ was increased by 5.2% by salbutamol (i.e. Post-drug level is 105.2% = 3.41 L/ 3.24 L). Post-drug to Post-exercise decline is 4.0% of the 105.2%. Thus FEV$_1$ decline is<br>-3.8% (= -4.0%/105.2%) from the Pre-drug level.<br>*Placebo:*<br>FEV$_1$ was not changed by placebo. Post-drug to Post-exercise decline is<br>-27.1%, which is also Pre-drug to Post-exercise change.			
Patel (1986)	Salbutamol 200 µg<br>Fig 2: 20 min test	9	-5.6%	-27.5%
Patessio (1991)	Salbutamol 200 µg<br>Fig 1: 2 hour test (1st test)	12	-8.2%	-24.8%
Pearlman (2006)	Albuterol 180 µg<br>Table 3: 15 min test	21	-3.52%	-11.11%
Pearlman (2007)	IPD	15		
Philip (2007)	Salmeterol 50 µg<br>Table 2: 2 hour test	46	-10.2%	-21.8%
Richter (2002)	Salmeterol 50 µg<br>Table 3: 30 min test	25	-7.6%	-22.4%
Shapiro (2002)	Albuterol 180 µg<br>Table II: 15 min test	17	-10.0%	-31.1%
Sturani (1983)	Salbutamol 200 µg<br>Fig 1B: 30 min test<br>Pre-drug as baseline	12	-11.8%	-31.9%
VanHalstma (2010)	Albuterol 180 µg<br>Fig 1, Caffeine 0 mg/kg: 15 min test	10	-4.1%	-14.4%
Walker (1986)	IPD	12		
Wolley (1990)	Terbutaline 500 µg<br>Fig 2: 15 min test<br>Pre-drug as baseline	12	-16%	-33.9%

Table S3: Calculation of the absolute and relative effects for Fig. 5: the Anderson (2001) trial as an example

**A:** Absolute effect of β_2-agonists					
	Placebo	β_2-Agonist	Effect		95% CI
	Mean	Mean	Absolute difference	SE	
	-39.4%	-11.0%	28.4 pp	3.0 pp	22.5 - 34.3 pp
**B:** Relative effect of β_2-agonists: Transformation to the relative scale by dividing by placebo test FEV_1 decline					
	Placebo	β_2-Agonist	Effect		95% CI
	Mean	Mean	Relative difference	SE	
	-1.0	-0.28	0.72	0.076	0.57 - 0.87
**C:** Relative effect of β_2-agonists from the slope of linear regression					
			Effect		95% CI
			Slope	SE	
			0.71	0.048	0.62 – 0.80

This table demonstrates the calculation of the 95% CIs for the three forest plots of Fig. 5.
The results shown are for the Anderson (2001) trial.

A: The absolute effect of β_2-agonists is calculated as the difference in the effects on the placebo and β_2-agonist tests, and the SE for the difference is calculated from the individual paired differences of the cross-over trial.

B: The relative effect is calculated by the transformation to the relative scale by dividing by the placebo test FEV_1 decline. Thus, on this scale, the effect of β_2-agonist is 72% reduction in the FEV_1 decline (based on 0.72 = 28.4/39.4), and the SE for that relative effect estimate is 7.6 pp (based on 0.076 = 3.0/39.4).

C: As a second method, the relative effect was calculated by linear regression, forcing the line though the origin, similar to Fig. 2 in the report, but restricting to the Anderson (2001) trial.
The slope of 0.71 has SE 0.048, corresponding to 71% effect with SE of 4.8 pp, see Additional File 1 for the calculation.
In each of the three scales, the 95% CI was calculated as the effect ± 1.96×SE. Therefore, each confidence interval is symmetric on the scale shown in Fig. 5.

Data extraction inconsistencies and errors in Bonini et al. (2013)

Our study did not intend to reproduce Bonini's main meta-analysis which was labeled Analysis 1.1 in their paper [11]. There are some errors and inaccuracies in the data extraction by Bonini and therefore exact reproduction of their Analysis 1.1 is not possible or relevant. Table S4 below describes the differences between Bonini's data extraction and ours.

Some of the errors are particularly large. In the Bronsky (1995) and the Del Col (1993) trials, Bonini added 10 and 20 percentage points to the published FEV_1 declines in the β_2-agonist tests, see below.

In particular, given that the effect of β_2-agonists decreases over time, for included studies that reported on exercise tests at various times after the administration of the β_2-agonist, we chose the shortest reported time after β_2-agonist administration. Of the 44 studies we included in our analysis, 39 (87%) published data of exercise test that was carried out within 1 hour after drug administration, and the others were carried out within 3 hours, except Carlsen (1995) which reported only the 10-12 hour exercise test.

As an example of misleading data extraction by Bonini [11], Kemp (1994) compared salbutamol and salmeterol in three exercise tests that were carried out 0.5, 5.5, and 11.5 hours after the administration of the β_2-agonist. In each time point, the FEV_1 decline was smaller after salmeterol than after salbutamol: 5% vs. 7% declines in the 0.5 hour test, 8% vs. 25% in the 5.5 hour test, and 13% vs. 27% in the 11.5 hour test, respectively. This means that at each time point salmeterol had a greater effect than salbutamol. However, in their Appendix 3, Bonini extracted the salbutamol FEV_1 decline from the 0.5 hour test (i.e. 7% FEV_1 decline) but the salmeterol FEV_1 decline from the 11.5 hour test (i.e. 13% FEV_1 decline) and thereby gives a biased impression that salbutamol was better than salmeterol because a smaller FEV_1 decline occurred after salbutamol. Such different time points were selected also for many other β_2-agonist comparisons, see below. Such arbitrary selection of exercise test times biases the presentation and analysis in the Bonini review.

The percentage decline in FEV_1 values in Table S3 indicate the change that occurred in the exercise test. The changes are negative, but the minus sign is not included.

For the references to the studies and to a description of the studies,
see Bonini [11]:
https://doi.org/10.1002/14651858.CD003564.pub3
https://www.ncbi.nlm.nih.gov/pubmed/24089311

Table S4: Data extraction inconsistencies and errors in Bonini et al. (2013)

Study	Original report<br>Source in the report	Bonini et al. [10] stated<br>Appendix 3: Raw data for the maximal percent fall in FEV_1 calculations
Blake (1999)	FEV_1 decline:<br>5.36% (Salmeterol 25) [1 hr test]<br>5.64% (Salmeterol 50) [1 hr test]<br>13.5% (Placebo) [1 hr test]<br>Table 3: 1 hour exercise test	FEV_1 decline:<br>7.99% (Salm 25) [6 hr test]<br>7.34% (Salm 50) [6 hr test]<br>14.0% (Placebo) [12 hr test]<br>Given that the effect of β_2-agonists decreases over time, we used the 1 hour exercise tests.<br>Furthermore, Bonini used different exercise test data for placebo and salmeterol.<br>Furthermore, for salbutamol (Albuterol) and its placebo, Bonini gives the 1 hour exercise test results (3.8% and 13.5%, respectively).<br>The same exercise test time should be used in the comparisons of the placebo and the β_2-agonists.
Bronsky (1995)	FEV_1 decline:<br>6% (Albuterol Aerosol)<br>6% (Albuterol Powder)<br>Table 1	FEV_1 decline:<br>16.0% (Salb MDI)<br>26.0% (Salb Pwd)<br>10% and 20% have been added in error by Bonini to the published results.
Bronsky (1999)	FEV_1 decline:<br>1.4% (Salmeterol Diskus) [1 hr]<br>0% (Salmeterol Diskhaler) [1 hr]<br>10.5% (Placebo) [1 hr]<br>Fig 1 and text p. 503: 1 hr exercise test	FEV_1 decline:<br>5.6% (Salm Disk) [12 hr test]<br>5.7% (Salm Diskhal) [6 hr test]<br>12.1% (Placebo) [12 hr test]<br>Given that the effect of β_2-agonists decreases over time, we used the 1 hour exercise tests.
Bronsky (2002)	FEV_1 decline:<br>~6% (Formoterol 12 μg) [15 min]<br>~6% (Formoterol 24 μg) [15 min]<br>Fig 1: 15 min after dosing	FEV_1 decline:<br>17.0% (Form 12 μg) [12 hr test]<br>14.6% (Form 24 μg) [12 hr test]<br>Given that the effect of β_2-agonists decreases over time, we used the 15 min tests.
Daugbjerg (1996)	FEV_1 decline:<br>9% (Formoterol) [3 hr test]<br>Page 685 bottom.<br>3 hour exercise test.<br>This 9% is reported as "median" in the original report.	FEV_1 decline:<br>11% (Form 12) [12 hr test]<br>Given that the effect of β_2-agonists decreases overtime, we used the 3 hour test.

**Study**	**Original report**<br>**Source in the report**	**Bonini et al. [10] stated**<br>**Appendix 3: Raw data for the maximal percent fall in FEV_1 calculations**
De Benedictis (1996)	FEV_1 decline:<br>10% (Salmeterol 25) [1 hr test]<br>4% (Salmeterol 50) [1 hr test]<br>Table 2: 1 hour exercise test	FEV_1 decline:<br>19.0% (Salm 25) [12 hr test]<br>15.0% (Salm 50) [12 hr test]<br>35.0% (Placebo) [1 hr test]<br>Given that the effect of β_2-agonists decreases over time, we used the 1 hour tests. The 1 hour test indicates substantially greater efficacy of salmeterol since the FEV_1 declines are much smaller.<br>Furthermore, Bonini used different exercise test data for placebo and salmeterol. The same exercise test time should be used in the comparison of placebo and β_2-agonist.
Del Col (1993)	FEV_1 decline:<br>0.76% (Albuterol + Jet)<br>2.37% (Albuterol + MDI)<br>Table 3	FEV_1 decline:<br>20.76% (Salb Jet)<br>12.37% (Salb MDI)<br>20% and 10% have been added in error by Bonini to the published results.
Green (1992)	FEV_1 decline:<br>2.7% (Salmeterol) [1 hr test]<br>Table 1: 1 hour exercise test.	FEV_1 decline:<br>3.2% (Salm 50) [9 hr test]<br>Given that the effect of β_2-agonists decreases over time, we used the 1 hour exercise test, though the difference is not great in this case.<br>Furthermore, the exact FEV_1 decline in the 9 hr test reported by Green (1992) was 3.4% and not the 3.2% stated by Bonini.
Grönneröd (2000)	FEV_1 decline:<br>5.40% (Formoterol 4.5 μg) [15 min]<br>2.50% (Formoterol 9 μg) [15 min]<br>18.4% (Placebo) [15 min test]<br>Table 2: 15 min exercise test	FEV_1 decline:<br>9.2% (Form 4.5) [12 hr test]<br>5.4% (Form 9) [12 hr test]<br>18.4% (Placebo) [15 min test]<br>Given that the effect of β_2-agonists decreases over time, we used the 15 min tests.<br>For placebo, Bonini gives the FEV_1 decline in the 15 min exercise test, but for formoterol results, Bonini seems to give the FEV_1 decline in the 12 hour exercise tests (9.29% and 5.43%) rounded down.
Kemp (1994)	FEV_1 decline:	FEV_1 decline:

**Study**	**Original report**<br>**Source in the report**	**Bonini et al. [10] stated**<br>**Appendix 3: Raw data for the maximal percent fall in FEV_1 calculations**
	5% (Salmeterol) [0.5 hr test]<br>7% (Albuterol) [0.5 hr test]<br>27% (Placebo) [0.5 hr test]<br>Table 2: 0.5 hour exercise test<br><br>We did not include the Kemp study in our analysis, since it was not a cross-over study.	13.0% (Salm) [11.5 hr test]<br>7.0% (Salb) [0.5 hr test]<br>27.0% (Placebo) [0.5 hr test]<br>Given that the effect of β_2-agonists decreases over time, we used the 0.5 hr tests.<br>For the parallel test on salbutamol (Albuterol), Bonini gives the FEV_1 decline in the 0.5 hour exercise test, but the salmeterol FEV_1 decline is from the 11.5 hour exercise test.<br>In the 0.5 hour test of salmeterol, the FEV_1 decline is 5%, which is smaller than the decline in the 0.5 hour test of salbutamol (i.e. 7%), see left-hand side.<br>Bonini's selection of the 0.5 hour exercise test for salbutamol and the 11.5 hour test for salmeterol misleads readers since the FEV_1 decline is greater on salmeterol treatment indicating that salmeterol is less effective. However, on each of the three reported time points, salmeterol was more effective in preventing FEV_1 decline.
König (1981)	FEV_1 decline for Study 1 is reported in Table 1 of König (1981)	Bonini does not include Study 1 results.<br>Study 1 had 24 participants; of these 24 participants, 17 participated in study 2, for which Bonini gives the results.<br>Study 1 had 10 min delay between inhaled metaproterenol and the exercise test.<br>Study 2 had 1 hr delay between inhaled metaproterenol and the exercise test.<br>Bonini writes as if there was a single trial which used two exercise tests "Time of exercise challenge after drug administration: 10 min, 1 hour" whereas König carried out two separate studies which used 10 min and 1 hour delay before the exercise tests.
McAlpine (1990)	FEV_1 decline:<br>14.1% (Salbutamol) [2 hr test]<br>Table 1: 2 hour exercise test	For the other studies, Bonini gives the results for all published β_2-agonists. For the McAlpine (1990) study, Bonini gives the formoterol results, but not the salbutamol results published in the same table.
Newnham (1993)	FEV_1 decline:<br>~1% (Salmeterol) [1 hr test]<br>27.1% (Placebo) [1 hr test]<br>Fig 1 and text p. 441	FEV_1 decline:<br>12.8% (Salmeterol) [12 hr test]<br>32.0% (Placebo) [6 hr test]<br>Given that the effect of β_2-agonists decreases over time, we used the 1 hour tests.<br>Furthermore, Bonini used different exercise test data for placebo and salmeterol.
Pearlman (2006)	FEV_1 decline:<br>2.61% (Formoterol 12 µg) [15 min]	FEV_1 decline:<br>7.6% (Form 12) [12 hr test]

Study	Original report<br>Source in the report	Bonini et al. [10] stated<br>Appendix 3: Raw data for the maximal percent fall in FEV_1 calculations
	1.02% (Formoterol 24 µg) [15 min]<br>11.11% (Placebo) [15 min test]<br>Table 3: 15 min exercise test	5.9% (Form 24) [12 hr test]<br>13.2% (Placebo) [4 hr test]<br>Given that the effect of β_2-agonists decreases over time, we used the 15 min tests.<br>Furthermore, Bonini used different exercise test data for placebo and formoterol. The same exercise test time should be used in the comparison of the placebo and the β_2-agonists.
Philip (2007)	FEV_1 decline:<br>10.2% (Salmeterol) [2 hr test]<br>Table 2: 2 hour exercise test	FEV_1 decline:<br>10.7% (Salm 50) [8.5 hr test]<br>21.8% (Placebo) [2 hr test]<br>Given that the effect of β_2-agonists decreases in time, we used the 2 hour tests.<br>Furthermore, Bonini used different exercise test data for placebo and salmeterol.
Richter (2002)	FEV_1 decline:<br>6.3% (Terbutaline) [30 min test]<br>22.4% (Placebo) [30 min test]<br>Table 3: 30 min exercise test	FEV_1 decline:<br>8.50% (Terb 500) [60 min test]<br>25.1% (Placebo) [60 min test]<br>For the parallel tests on formoterol and salmeterol, Bonini gives the FEV_1 declined in the 30 min exercise tests (5.7% and 7.6%, respectively), but for the terbutaline and placebo FEV_1 declines they give the results from the 60 min exercise test.<br>The same time exercise test should be used in the comparison of placebo and β_2-agonist.
Shapiro GS (2002)	FEV_1 decline:<br>4.0% (Formoterol 12 µg) [15 min]<br>6.0% (Formoterol 24 µg) [15 min]<br>Table II: 15 min exercise test	FEV_1 decline:<br>12.4% (Form 12) [12 hr test]<br>17.5% (Form 24) [12 hr test]<br>10.0% (Salb 180) [15 min test]<br>Given that the effect of β_2-agonists decreases over time, we used the 15 min tests.<br>For salbutamol, Bonini gives the FEV_1 decline in the 15 min exercise test. Thereby the comparison with formoterol (i.e. 12 hr test) is biased and gives an impression that salbutamol is better, though formoterol is better in both the 15 min and the 12 hr tests when compared with salbutamol at the same time points.<br>The same time exercise test should be used in the comparison of placebo and β_2-agonists.<br>Finally, Bonini's reference is erroneous, to a paper by a different Shapiro GG (1990): https://www.ncbi.nlm.nih.gov/pubmed/2145791 and not to the Shapiro GS (2002) though Bonini's data are from the 2002 paper: https://www.ncbi.nlm.nih.gov/pubmed/12581546

Printouts of statistical calculations

Table 2 and Fig 2 calculations

```
> BetaLmerI <- lmer(Beta$Difference ~  1 + (1|Beta$Type:Beta$Study))
> summary(BetaLmerI)
Linear mixed model fit by REML ['lmerMod']
Formula: Beta$Difference ~ 1 + (1 | Beta$Type:Beta$Study)


Random effects:
 Groups               Name        Variance Std.Dev.
 Beta$Type:Beta$Study (Intercept)  83.2     9.12
 Residual                         333.4    18.26
Number of obs: 187, groups:  Beta$Type:Beta$Study, 14

Fixed effects:
            Estimate Std. Error t value
(Intercept)     27.7        2.8    9.91
> confint(BetaLmerI)
Computing profile confidence intervals ...
              2.5 % 97.5 %
.sig01       5.1007 14.493
.sigma      16.4920 20.364
(Intercept) 22.0950 33.436

> BetaLmer <- lmer(Beta$Difference ~  Beta$Placebo + (Beta$Placebo|
Beta$Type:Beta$Study))
> summary(BetaLmer)
Linear mixed model fit by REML ['lmerMod']
Formula: Beta$Difference ~ Beta$Placebo + (Beta$Placebo | Beta$Type:Beta$Study)


Random effects:
 Groups               Name         Variance Std.Dev. Corr
 Beta$Type:Beta$Study (Intercept)   60.0372  7.748
                      Beta$Placebo   0.0895  0.299   0.50
 Residual                          200.8788 14.173
Number of obs: 187, groups:  Beta$Type:Beta$Study, 14

Fixed effects:
             Estimate Std. Error t value
(Intercept)     7.907      3.080    2.57
Beta$Placebo   -0.691      0.106   -6.54

Correlation of Fixed Effects:
            (Intr)
Beta$Placeb 0.663
> confint(BetaLmer)
Computing profile confidence intervals ...
                 2.5 %   97.5 %
.sig01        0.459740 14.40500
.sig02       -1.000000  0.68553
.sig03        0.094367  0.51754
.sigma       12.759799 15.85445
(Intercept)   1.850442 14.49775
Beta$Placebo -0.909915 -0.47769
There were 50 or more warnings (use warnings() to see the first 50)
>
> BetaLmerS <- lmer(Beta$Difference ~  Beta$Placebo -1+ (Beta$Placebo -1|
Beta$Type:Beta$Study))
> summary(BetaLmerS)
Linear mixed model fit by REML ['lmerMod']
Formula: Beta$Difference ~ Beta$Placebo - 1 + (Beta$Placebo - 1 |
Beta$Type:Beta$Study)
```

```
REML criterion at convergence: 1570.8

Scaled residuals:
    Min      1Q  Median      3Q     Max
-3.464 -0.227  0.135  0.511  4.545

Random effects:
 Groups               Name         Variance Std.Dev.
 Beta$Type:Beta$Study Beta$Placebo   0.0916  0.303
 Residual                          223.2708 14.942
Number of obs: 187, groups:  Beta$Type:Beta$Study, 14

Fixed effects:
             Estimate Std. Error t value
Beta$Placebo  -0.8975     0.0898     -10
> confint(BetaLmerS)
Computing profile confidence intervals ...
                 2.5 %   97.5 %
.sig01         0.18082  0.47730
.sigma        13.49674 16.67375
Beta$Placebo -1.08562 -0.71924
```

```
> anova(BetaLmerI,BetaLmer)
refitting model(s) with ML (instead of REML)
Data: NULL
Models:
BetaLmerI: Beta$Difference ~ 1 + (1 | Beta$Type:Beta$Study)
BetaLmer: Beta$Difference ~ Beta$Placebo + (Beta$Placebo |
Beta$Type:Beta$Study)
          Df  AIC  BIC logLik deviance Chisq Chi Df Pr(>Chisq)
BetaLmerI  3 1642 1652   -818     1636
BetaLmer   6 1566 1585   -777     1554  82.3      3     <2e-16 ***
---

> anova(BetaLmer,BetaLmerS)
refitting model(s) with ML (instead of REML)
Data: NULL
Models:
BetaLmerS: Beta$Difference ~ Beta$Placebo - 1 + (Beta$Placebo - 1 |
Beta$Type:Beta$Study)
BetaLmer: Beta$Difference ~ Beta$Placebo + (Beta$Placebo |
Beta$Type:Beta$Study)
          Df  AIC  BIC logLik deviance Chisq Chi Df Pr(>Chisq)
BetaLmerS  3 1574 1583   -784     1568
BetaLmer   6 1566 1585   -777     1554  13.9      3      0.003 **
---

> AIC(BetaLmerI,BetaLmerS)
          df    AIC
BetaLmerI  3 1638.3
BetaLmerS  3 1576.8


> median(abs(residuals(BetaLmerI)))
[1] 10.829
> median(abs(residuals(BetaLmerS)))
[1] 5.8229
```

Table 3 calculations

```
> Beta$Pl_10 <- Beta$Placebo<= -10&Beta$Placebo> -20
> Beta10 <- Beta[Beta$Pl_10 ==1,]
> Beta_10 <- lmer(Beta10$Difference ~  1 + (1|Beta10$Type:Beta10$Study))
> summary(Beta_10)
Linear mixed model fit by REML ['lmerMod']
Formula: Beta10$Difference ~ 1 + (1 | Beta10$Type:Beta10$Study)

REML criterion at convergence: 235.3

Random effects:
 Groups                    Name        Variance Std.Dev.
 Beta10$Type:Beta10$Study (Intercept) 45.71    6.761
 Residual                             57.05    7.553
Number of obs: 33, groups:  Beta10$Type:Beta10$Study, 12

Fixed effects:
            Estimate Std. Error t value
(Intercept)   15.224      2.465   6.176
> confint(Beta_10)
Computing profile confidence intervals ...
                2.5 %   97.5 %
.sig01       2.236469 11.92368
.sigma       5.749707 10.45168
(Intercept) 10.225639 20.29275
>
> length(Beta10$Placebo)
[1] 33
> mean(Beta10$Placebo)
[1] -15.49694
> sd(Beta10$Placebo)
[1] 2.674083
> mean(Beta10$Difference)
[1] 15.32273
> sd(Beta10$Difference)
[1] 10.00691
>
>
> Beta$Pl_20 <- Beta$Placebo<= -20&Beta$Placebo> -30
> Beta20 <- Beta[Beta$Pl_20 ==1,]
> Beta_20 <- lmer(Beta20$Difference ~  1 + (1|Beta20$Type:Beta20$Study))
> summary(Beta_20)
Linear mixed model fit by REML ['lmerMod']
Formula: Beta20$Difference ~ 1 + (1 | Beta20$Type:Beta20$Study)

REML criterion at convergence: 225.5

Random effects:
 Groups                    Name        Variance Std.Dev.
 Beta20$Type:Beta20$Study (Intercept)  45.41    6.739
 Residual                             129.74   11.390
Number of obs: 29, groups:  Beta20$Type:Beta20$Study, 12

Fixed effects:
            Estimate Std. Error t value
(Intercept)   23.620      2.947   8.015
> confint(Beta_20)
Computing profile confidence intervals ...
                2.5 %   97.5 %
.sig01       0.000000 12.95123
.sigma       8.547118 15.90304
(Intercept) 17.766230 29.86248
>
> length(Beta20$Placebo)
[1] 29
```

```
> mean(Beta20$Placebo)
[1] -24.67914
> sd(Beta20$Placebo)
[1] 2.985238
> mean(Beta20$Difference)
[1] 22.59383
> sd(Beta20$Difference)
[1] 13.31154
>
>
> Beta$Pl_30 <- Beta$Placebo<= -30&Beta$Placebo> -40
> Beta30 <- Beta[Beta$Pl_30 ==1,]
> Beta_30 <- lmer(Beta30$Difference ~  1 + (1|Beta30$Type:Beta30$Study))
> summary(Beta_30)
Linear mixed model fit by REML ['lmerMod']
Formula: Beta30$Difference ~ 1 + (1 | Beta30$Type:Beta30$Study)

REML criterion at convergence: 290

Random effects:
 Groups                   Name        Variance Std.Dev.
 Beta30$Type:Beta30$Study (Intercept)  69.2     8.319
 Residual                             293.1    17.119
Number of obs: 34, groups:  Beta30$Type:Beta30$Study, 12

Fixed effects:
            Estimate Std. Error t value
(Intercept)   32.981      3.884   8.491
> confint(Beta_30)
Computing profile confidence intervals ...
               2.5 %   97.5 %
.sig01       0.00000 17.71278
.sigma      13.10969 23.43105
(Intercept) 25.03157 40.92001
>
> length(Beta30$Placebo)
[1] 34
> mean(Beta30$Placebo)
[1] -34.54618
> sd(Beta30$Placebo)
[1] 2.646329
> mean(Beta30$Difference)
[1] 32.81453
> sd(Beta30$Difference)
[1] 18.87488
>
>
> Beta$Pl_40 <- Beta$Placebo<= -40&Beta$Placebo> -50
> Beta40 <- Beta[Beta$Pl_40 ==1,]
> Beta_40 <- lmer(Beta40$Difference ~  1 + (1|Beta40$Type:Beta40$Study))
> summary(Beta_40)
Linear mixed model fit by REML ['lmerMod']
Formula: Beta40$Difference ~ 1 + (1 | Beta40$Type:Beta40$Study)

REML criterion at convergence: 242.1


Random effects:
 Groups                   Name        Variance Std.Dev.
 Beta40$Type:Beta40$Study (Intercept) 125.6    11.21
 Residual                             218.7    14.79
Number of obs: 29, groups:  Beta40$Type:Beta40$Study, 11

Fixed effects:
            Estimate Std. Error t value
(Intercept)   39.703      4.572   8.683
> confint(Beta_40)
```

```
Computing profile confidence intervals ...
                2.5 %   97.5 %
.sig01       2.822957 20.31467
.sigma      11.159536 20.52371
(Intercept) 30.588948 49.30061
>
> length(Beta40$Placebo)
[1] 29
> mean(Beta40$Placebo)
[1] -44.47872
> sd(Beta40$Placebo)
[1] 3.108381
> mean(Beta40$Difference)
[1] 36.85131
> sd(Beta40$Difference)
[1] 18.82314
>
>
>
> Beta$Pl_50 <- Beta$Placebo<= -50
> Beta50 <- Beta[Beta$Pl_50 ==1,]
> Beta_50 <- lmer(Beta50$Difference ~  1 + (1|Beta50$Type:Beta50$Study))
> summary(Beta_50)
Linear mixed model fit by REML ['lmerMod']
Formula: Beta50$Difference ~ 1 + (1 | Beta50$Type:Beta50$Study)

REML criterion at convergence: 294.6


Random effects:
 Groups                   Name        Variance Std.Dev.
 Beta50$Type:Beta50$Study (Intercept) 104.0    10.20
 Residual                             332.8    18.24
Number of obs: 34, groups:  Beta50$Type:Beta50$Study, 10

Fixed effects:
            Estimate Std. Error t value
(Intercept)   44.302      4.775   9.278
> confint(Beta_50)
Computing profile confidence intervals ...
               2.5 %   97.5 %
.sig01       0.00000 20.76483
.sigma      14.16507 24.91224
(Intercept) 34.82413 54.63546
>
>
> length(Beta50$Placebo)
[1] 34
> mean(Beta50$Placebo)
[1] -59.85812
> sd(Beta50$Placebo)
[1] 7.317866
> mean(Beta50$Difference)
[1] 42.50647
> sd(Beta50$Difference)
[1] 20.43452
```

Fig 3 calculations

```
> BetaOver10 <- Beta[Beta$Placebo<=-10,]
>
>
> skewness(BetaOver10$Relative)
[1] -1.053634


>
>
> iqr=c(0.25, 0.5, 0.75)
> BetaQR <- rq(Beta$Difference ~  Beta$Placebo -1, tau = iqr)
> summary(BetaQR)

Call: rq(formula = Beta$Difference ~ Beta$Placebo - 1, tau = iqr)

tau: [1] 0.25

Coefficients:
Beta$Placebo
        -0.6

Call: rq(formula = Beta$Difference ~ Beta$Placebo - 1, tau = iqr)

tau: [1] 0.5

Coefficients:
Beta$Placebo
    -0.88462

Call: rq(formula = Beta$Difference ~ Beta$Placebo - 1, tau = iqr)

tau: [1] 0.75

Coefficients:
Beta$Placebo
    -1.03129
 >
```

Table 4 and Fig 4: All the 44 trials

```
> MeansLmerI <- lmer(Means$Difference ~ 1  + (1|Means$Type), weights =Means$N)
> summary(MeansLmerI)
Linear mixed model fit by REML ['lmerMod']
Formula: Means$Difference ~ 1 + (1 | Means$Type)
Weights: Means$N



Random effects:
 Groups     Name        Variance Std.Dev.
 Means$Type (Intercept)    5.28   2.3    
 Residual               1341.48  36.6    
Number of obs: 44, groups:  Means$Type, 9

Fixed effects:
            Estimate Std. Error t value
(Intercept)    21.42       1.76    12.2
>
> MeansLmer <- lmer(Means$Difference ~ Means$Placebo + (Means$Placebo|
Means$Type), weights =Means$N)
Warning message:
In checkConv(attr(opt, "derivs"), opt$par, ctrl = control$checkConv,  :
  Model failed to converge with max|grad| = 0.00455223 (tol = 0.002, component
1)
> summary(MeansLmer)
Linear mixed model fit by REML ['lmerMod']
Formula: Means$Difference ~ Means$Placebo + (Means$Placebo | Means$Type)
Weights: Means$N



Random effects:
 Groups     Name          Variance Std.Dev. Corr
 Means$Type (Intercept)    157.461 12.548       
            Means$Placebo    0.123  0.351   1.00
 Residual                 1021.172 31.956       
Number of obs: 44, groups:  Means$Type, 9

Fixed effects:
              Estimate Std. Error t value
(Intercept)     16.426      6.970    2.36
Means$Placebo   -0.241      0.210   -1.15

Correlation of Fixed Effects:
            (Intr)
Means$Placb 0.975 
convergence code: 0
Model failed to converge with max|grad| = 0.00455223 (tol = 0.002, component 1)

>
> MeansLmerS <- lmer(Means$Difference ~ Means$Placebo -1 + (Means$Placebo-1|
Means$Type), weights =Means$N)
> summary(MeansLmerS)
Linear mixed model fit by REML ['lmerMod']
Formula: Means$Difference ~ Means$Placebo - 1 + (Means$Placebo - 1 |
Means$Type)
Weights: Means$N



Random effects:
 Groups     Name          Variance Std.Dev.
 Means$Type Means$Placebo    0      0.0    
 Residual                 1489     38.6    
Number of obs: 44, groups:  Means$Type, 9

Fixed effects:
              Estimate Std. Error t value
```

```
Means$Placebo  -0.7662     0.0504   -15.2
>
> anova(MeansLmer,MeansLmerI)
refitting model(s) with ML (instead of REML)
Data: NULL
Models:
MeansLmerI: Means$Difference ~ 1 + (1 | Means$Type)
MeansLmer: Means$Difference ~ Means$Placebo + (Means$Placebo | Means$Type)
           Df AIC BIC logLik deviance Chisq Chi Df Pr(>Chisq)
MeansLmerI  3 326 331   -160      320
MeansLmer   6 323 334   -156      311  8.52      3      0.036 *
---

> anova(MeansLmer,MeansLmerS)
refitting model(s) with ML (instead of REML)
Data: NULL
Models:
MeansLmerS: Means$Difference ~ Means$Placebo - 1 + (Means$Placebo - 1 |
Means$Type)
MeansLmer: Means$Difference ~ Means$Placebo + (Means$Placebo | Means$Type)
           Df AIC BIC logLik deviance Chisq Chi Df Pr(>Chisq)
MeansLmerS  3 329 334   -161      323
MeansLmer   6 323 334   -156      311  11.3      3       0.01 *
---

>
> AIC(MeansLmerI,MeansLmerS)
           df    AIC
MeansLmerI  3 323.49
MeansLmerS  3 332.96
```

Fig 5: Calculation of slope and its SE for each of the 14 studies with IPD

```
> And <- Beta[Beta$Study=="Anderson2001",]
> summary(AndLm <- lm(And$Difference ~  And$Placebo- 1 ))

Call:
lm(formula = And$Difference ~ And$Placebo - 1)

Coefficients:
            Estimate Std. Error t value Pr(>|t|)
And$Placebo -0.70931    0.04783  -14.83 3.37e-14 ***
---

Residual standard error: 10.69 on 26 degrees of freedom
Multiple R-squared:  0.8943,    Adjusted R-squared:  0.8902
F-statistic: 219.9 on 1 and 26 DF,  p-value: 3.37e-14

> mean(And$bAgon)
[1] -10.96852
> sd(And$bAgon)
[1] 12.31758
> mean(And$Placebo)
[1] -39.4037
> sd(And$Placebo)
[1] 17.57891
>
>
> Bon <- Beta[Beta$Study=="Boner1994",]
> summary(lm(Bon$Difference ~  Bon$Placebo- 1 ))

Call:
lm(formula = Bon$Difference ~ Bon$Placebo - 1)

Coefficients:
            Estimate Std. Error t value Pr(>|t|)
Bon$Placebo  -0.4562     0.1970  -2.316   0.0362 *
---

Residual standard error: 14.81 on 14 degrees of freedom
Multiple R-squared:  0.277,     Adjusted R-squared:  0.2253
F-statistic: 5.363 on 1 and 14 DF,  p-value: 0.03624

> mean(Bon$bAgon)
[1] -9.533333
> sd(Bon$bAgon)
[1] 15.53276
> mean(Bon$Placebo)
[1] -14.46667
> sd(Bon$Placebo)
[1] 13.39438
>
>
```

```
> Deb <- Beta[Beta$Study=="Debelic1988",]
> summary(lm(Deb$Difference ~  Deb$Placebo- 1 ))

Call:
lm(formula = Deb$Difference ~ Deb$Placebo - 1)

Coefficients:
            Estimate Std. Error t value Pr(>|t|)
Deb$Placebo  -0.6289     0.1465  -4.294 0.000639 ***
---

Residual standard error: 25.51 on 15 degrees of freedom
Multiple R-squared:  0.5514,    Adjusted R-squared:  0.5215
F-statistic: 18.44 on 1 and 15 DF,  p-value: 0.0006393

> mean(Deb$bAgon)
[1] -12.6125
> sd(Deb$bAgon)
[1] 27.55965
> mean(Deb$Placebo)
[1] -38.54375
> sd(Deb$Placebo)
[1] 20.92268
>
>
> de96 <- Beta[Beta$Study=="de Benedictis 1996",]
> summary(lm(de96$Difference ~  de96$Placebo- 1 ))

Call:
lm(formula = de96$Difference ~ de96$Placebo - 1)

Coefficients:
             Estimate Std. Error t value Pr(>|t|)
de96$Placebo -0.90246    0.02504  -36.05 9.02e-13 ***
---

Residual standard error: 3.408 on 11 degrees of freedom
Multiple R-squared:  0.9916,    Adjusted R-squared:  0.9908
F-statistic:  1299 on 1 and 11 DF,  p-value: 9.02e-13

> mean(de96$bAgon)
[1] -4
> sd(de96$bAgon)
[1] 3.190896
> mean(de96$Placebo)
[1] -36.33333
> sd(de96$Placebo)
[1] 15.62244
>
>
```

```
> de98 <- Beta[Beta$Study=="de Benedictis 1998",]
> summary(lm(de98$Difference ~  de98$Placebo- 1 ))

Call:
lm(formula = de98$Difference ~ de98$Placebo - 1)

Coefficients:
             Estimate Std. Error t value Pr(>|t|)
de98$Placebo -0.90489    0.04605  -19.65 6.46e-10 ***
---

Residual standard error: 5.022 on 11 degrees of freedom
Multiple R-squared:  0.9723,    Adjusted R-squared:  0.9698
F-statistic: 386.1 on 1 and 11 DF,  p-value: 6.464e-10

> mean(de98$bAgon)
[1] -3.75
> sd(de98$bAgon)
[1] 4.433857
> mean(de98$Placebo)
[1] -25.75
> sd(de98$Placebo)
[1] 18.91188
>
>
> Din <- Beta[Beta$Study=="Dinh Xuan 1989",]
> summary(lm(Din$Difference ~  Din$Placebo- 1 ))

Call:
lm(formula = Din$Difference ~ Din$Placebo - 1)

Coefficients:
            Estimate Std. Error t value Pr(>|t|)
Din$Placebo -0.94646    0.07758   -12.2 6.69e-07 ***
---

Residual standard error: 10.96 on 9 degrees of freedom
Multiple R-squared:  0.943,     Adjusted R-squared:  0.9366
F-statistic: 148.8 on 1 and 9 DF,  p-value: 6.688e-07

> mean(Din$bAgon)
[1] 0.0509
> sd(Din$bAgon)
[1] 11.25025
> mean(Din$Placebo)
[1] -41.0796
> sd(Din$Placebo)
[1] 18.55219
>
>
```

```
> Gre <- Beta[Beta$Study=="Green1992",]
> summary(lm(Gre$Difference ~  Gre$Placebo- 1 ))

Call:
lm(formula = Gre$Difference ~ Gre$Placebo - 1)


Coefficients:
            Estimate Std. Error t value Pr(>|t|)    
Gre$Placebo   -1.588      0.127   -12.5 3.06e-08 ***
---

Residual standard error: 11.65 on 12 degrees of freedom
Multiple R-squared:  0.9287,    Adjusted R-squared:  0.9228 
F-statistic: 156.3 on 1 and 12 DF,  p-value: 3.057e-08 

> mean(Gre$bAgon)
[1] 14.35285
> sd(Gre$bAgon)
[1] 12.46346
> mean(Gre$Placebo)
[1] -21.17462
> sd(Gre$Placebo)
[1] 14.69036
> 
> 
> He83 <- Beta[Beta$Study=="Henriksen1983",]
> summary(lm(He83$Difference ~  He83$Placebo- 1 ))

Call:
lm(formula = He83$Difference ~ He83$Placebo - 1)


Coefficients:
             Estimate Std. Error t value Pr(>|t|)   
He83$Placebo -0.29063    0.08418  -3.452  0.00429 **
---

Residual standard error: 15.3 on 13 degrees of freedom
Multiple R-squared:  0.4783,    Adjusted R-squared:  0.4382 
F-statistic: 11.92 on 1 and 13 DF,  p-value: 0.004289 

> mean(He83$bAgon)
[1] -30.50721
> sd(He83$bAgon)
[1] 22.57338
> mean(He83$Placebo)
[1] -46.29679
> sd(He83$Placebo)
[1] 15.20486
> 
> 
> 
```

```
> He92 <- Beta[Beta$Study=="Henriksen1992",]
> summary(lm(He92$Difference ~  He92$Placebo- 1 ))

Call:
lm(formula = He92$Difference ~ He92$Placebo - 1)

Coefficients:
             Estimate Std. Error t value Pr(>|t|)
He92$Placebo  -0.9344     0.1522  -6.141  7.3e-05 ***
---

Residual standard error: 24.16 on 11 degrees of freedom
Multiple R-squared:  0.7742,    Adjusted R-squared:  0.7536
F-statistic: 37.71 on 1 and 11 DF,  p-value: 7.303e-05

> mean(He92$bAgon)
[1] -1.165583
> sd(He92$bAgon)
[1] 24.33675
> mean(He92$Placebo)
[1] -43.75
> sd(He92$Placebo)
[1] 14.29638
>
>
>
> Pea <- Beta[Beta$Study=="Pearlman2007",]
> summary(lm(Pea$Difference ~  Pea$Placebo- 1 ))

Call:
lm(formula = Pea$Difference ~ Pea$Placebo - 1)

Coefficients:
            Estimate Std. Error t value Pr(>|t|)
Pea$Placebo -0.96651    0.02315  -41.75 4.29e-16 ***
---

Residual standard error: 2.304 on 14 degrees of freedom
Multiple R-squared:  0.992,     Adjusted R-squared:  0.9915
F-statistic:  1743 on 1 and 14 DF,  p-value: 4.287e-16

> mean(Pea$bAgon)
[1] -1.306667
> sd(Pea$bAgon)
[1] 2.06725
> mean(Pea$Placebo)
[1] -21.76
> sd(Pea$Placebo)
[1] 14.15197
>
>
>
```

```
> Rob <- Beta[Beta$Study=="Robertson1994",]
> summary(lm(Rob$Difference ~  Rob$Placebo- 1 ))

Call:
lm(formula = Rob$Difference ~ Rob$Placebo - 1)

Coefficients:
            Estimate Std. Error t value Pr(>|t|)
Rob$Placebo  -1.5855     0.4845  -3.273   0.0136 *
---

Residual standard error: 16.47 on 7 degrees of freedom
Multiple R-squared:  0.6048,    Adjusted R-squared:  0.5483
F-statistic: 10.71 on 1 and 7 DF,  p-value: 0.01362

> mean(Rob$bAgon)
[1] 12.0215
> sd(Rob$bAgon)
[1] 12.75419
> mean(Rob$Placebo)
[1] -8.32975
> sd(Rob$Placebo)
[1] 9.262858
>
>
> Sch <- Beta[Beta$Study=="Schoeffel1981",]
> summary(lm(Sch$Difference ~  Sch$Placebo- 1 ))

Call:
lm(formula = Sch$Difference ~ Sch$Placebo - 1)

Coefficients:
            Estimate Std. Error t value Pr(>|t|)
Sch$Placebo  -0.8397     0.0437  -19.21 1.29e-08 ***
---

Residual standard error: 5.438 on 9 degrees of freedom
Multiple R-squared:  0.9762,    Adjusted R-squared:  0.9736
F-statistic: 369.2 on 1 and 9 DF,  p-value: 1.293e-08

> mean(Sch$bAgon)
[1] -5.58
> sd(Sch$bAgon)
[1] 6.261842
> mean(Sch$Placebo)
[1] -37.56
> sd(Sch$Placebo)
[1] 12.36916
>
>
```

```
> Sim <- Beta[Beta$Study=="Simons1997",]
> summary(lm(Sim$Difference ~  Sim$Placebo- 1 ))

Call:
lm(formula = Sim$Difference ~ Sim$Placebo - 1)

Coefficients:
            Estimate Std. Error t value Pr(>|t|)
Sim$Placebo -1.29220    0.06678  -19.35 2.96e-09 ***
---

Residual standard error: 5.758 on 10 degrees of freedom
Multiple R-squared:  0.974,     Adjusted R-squared:  0.9714
F-statistic: 374.5 on 1 and 10 DF,  p-value: 2.961e-09

> mean(Sim$bAgon)
[1] 7.942636
> sd(Sim$bAgon)
[1] 5.218767
> mean(Sim$Placebo)
[1] -23.62709
> sd(Sim$Placebo)
[1] 11.37575
>
>
>
> Wal <- Beta[Beta$Study=="Walker1986",]
> summary(lm(Wal$Difference ~  Wal$Placebo- 1 ))

Call:
lm(formula = Wal$Difference ~ Wal$Placebo - 1)

Coefficients:
            Estimate Std. Error t value Pr(>|t|)
Wal$Placebo  -1.0832     0.2387  -4.537 0.000848 ***
---

Residual standard error: 26.13 on 11 degrees of freedom
Multiple R-squared:  0.6518,    Adjusted R-squared:  0.6201
F-statistic: 20.59 on 1 and 11 DF,  p-value: 0.0008476

> mean(Wal$bAgon)
[1] 11.14825
> sd(Wal$bAgon)
[1] 23.55104
> mean(Wal$Placebo)
[1] -26.16592
> sd(Wal$Placebo)
[1] 18.4925
```

Estimation of the possible role of the regression to the mean phenomenon

Approach 1

```
> str(Placebo)
'data.frame':  45 obs. of  4 variables:
 $ Study     : Factor w/ 4 levels "deBenedictis1996",..: 1 1 1 1 1 1 1 1 1
1 ...
 $ Placebo1  : num  35 48 30 35 19 61 19 60 27 54 ...
 $ Placebo2  : num  26 46 8 29 23 50 25 50 23 48 ...

>
> Placebo$Difference <- Placebo$Placebo2 -Placebo$Placebo1
>
> PlaceboLmer <- lmer(Placebo$Difference ~  Placebo$Placebo1 +
(Placebo$Placebo1|Placebo$Study))
> summary(PlaceboLmer)
Linear mixed model fit by REML ['lmerMod']
Formula: Placebo$Difference ~ Placebo$Placebo1 + (Placebo$Placebo1 |
Placebo$Study)



Random effects:
 Groups        Name             Variance     Std.Dev.     Corr
 Placebo$Study (Intercept)      2.517846e-06 1.586772e-03
               Placebo$Placebo1 6.591825e-09 8.119005e-05 -1.00000
 Residual                       7.007141e+01 8.370866e+00
Number of obs: 45, groups:  Placebo$Study, 4

Fixed effects:
                   Estimate  Std. Error  t value
(Intercept)       1.61510507  2.95396576  0.54676
Placebo$Placebo1 -0.15287875  0.08054546 -1.89804

Correlation of Fixed Effects:
            (Intr)
Placb$Plcb1 -0.906
```

Approach 2

```
> str(PlaceboLm)
'data.frame':   103 obs. of  3 variables:
 $ Study : Factor w/ 4 levels "de Benedictis 1996",..: 1 1 1 1 1 1 1 1 1 1 ...
 $ person: Factor w/ 45 levels "1","2","3","4",..: 1 2 3 4 5 6 7 8 9 10 ...
 $ FEV1  : num  35 48 30 35 19 61 19 60 27 54 ...
> Plac_Var4 <- lmer(PlaceboLm$FEV1 ~     1+ (1|PlaceboLm$person))
>
> Plac_Var4
Linear mixed model fit by REML ['lmerMod']
Formula: PlaceboLm$FEV1 ~ 1 + (1 | PlaceboLm$person)
REML criterion at convergence: 778.1398
Random effects:
 Groups           Name        Std.Dev.
 PlaceboLm$person (Intercept) 14.02300
 Residual                      6.23319    # SD based on mixed-effects model
Number of obs: 103, groups:  PlaceboLm$person, 45
Fixed Effects:
(Intercept)
   31.77804

> # Blomqvist formula calculation
>
> sd(Beta$Placebo)
[1] 18.878
>
> rho<- 1 - (6.23319^2/18.87793^2)
> rho
[1] 0.89098
>
> beta_observed <- -0.6911
>
> beta_true <- (beta_observed + 1 - rho)/rho
> beta_true
[1] -0.6533
>
> ratio_beta <- beta_true/beta_observed
> ratio_beta     #
[1] 0.94531
Only about 5% error due to regression to mean, which is small compared with the
width of the 95% CI of the slope
```

Placebo-test vs placebo test

```
> beta_observed <- -0.15288
>
> beta_true <- (beta_observed + 1 - rho)/rho
> beta_true  #  essentially all slope is due to regression to mean
[1] -0.049225
```
